# *Mytho/Phaf1* is required to prevent DNA damage and tissue degeneration in *Danio rerio*

**DOI:** 10.1038/s41420-026-03106-x

**Published:** 2026-04-17

**Authors:** Tommaso Pagliarusco, Anais Franco-Romero, Francesca Terrin, Filippo Citton, Ludovica Carducci, Nicola Facchinello, Camilla Maria Fontana, Marta Giacomazzo, Ranieri Verin, Maria Berica Rasotto, Lisa Locatello, Luisa Dalla Valle, Marco Sandri

**Affiliations:** 1 Department of Biomedical Sciences, University of Padova, Padova, Italy; 2 Veneto Institute of Molecular Medicine, Padova, Italy; 3 Department of Biology, University of Padova, Padova, Italy; 4 Department of Pharmacy and Biotechnology, University of Bologna, Bologna, Italy; 5 Neuroscience Institute, Italian Research Council (CNR), Padova, Italy; 6 Department of Comparative Biomedicine and Food Science (BCA), University of Padova, Legnaro, PD Italy; 7 Department of Veterinary Sciences, University of Pisa, Pisa, Italy; 8 Stazione Zoologica Anton Dohrn, Department of Biology and Evolution of Marine Organisms, Fano Marine Center, Fano, Italy; 9 Department of Medicine, McGill University, Montreal, QC Canada

**Keywords:** Macroautophagy, Infertility, Ageing

## Abstract

*mytho* (Macroautophagy and YouTH Optimizer) is a novel FoxO-dependent gene that has been recently identified to control health- and life-span in *Caenorhabditis* *elegans* via autophagy regulation. However, the role of this gene in tissues development and function in vertebrates has not yet been established. To address these issues, we generated a zebrafish *mytho* KO model and observed that mutants exhibited a higher mortality rate than wild-type (WT) siblings during the first month of life and a lower resistance to oxidative stress. *mytho* silencing resulted in a decrease in larval locomotor activity and muscle birefringence and caused alteration of adult muscle structure. Autophagy impairment was confirmed in tissues with the highest *mytho* expression such as brain, muscle and testis. Finally, mutants showed tissue degeneration in pancreas, retina and muscle, morphological alterations in gonads of both sexes and a reduction of reproductive capabilities of males. Importantly, males presented a higher incidence of seminomas, a testicular cancer. The increased susceptibility to cancer is associated with an enhanced DNA fragmentation in sperm cells. In conclusion, this study highlights the key role of Mytho in maintaining proper tissue function and DNA integrity.

## Introduction

Autophagy plays a fundamental role in maintenance of cellular homeostasis and tissues function. Indeed, the autophagy-lysosome system removes dysfunctional organelles and toxic proteins allowing the recycle of macromolecules for both bioenergetic and regenerative purposes. Numerous human diseases, including cancer, myopathies and neurodegeneration, are caused by autophagy-lysosome impairment. Reproductive disorders and infertility have also been linked to reduced autophagic flux [1–3], this system plays several functional roles in spermatogenesis, oogenesis, and development [4, 5].

In recent years, the understanding of the autophagic machinery has undergone a tremendous progress, thanks to the characterization of proteins that are crucial to the regulation of this system. We recently contributed to the field by identifying a novel protein called MYTHO/PHAF1, hereafter named MYTHO (Macroautophagy and YouTH Optimizer), that, by interacting with WIPI2, allows the recruitment of the conjugation machinery ATG16L1/ATG5/ATG12 to the PIP3-positive membranes at the phagophore initiation site, to promote LC3 lipidation, membrane commitment and autophagosome formation [6].

*Mytho* was identified by screening genes that were induced during enhanced protein breakdown in skeletal muscle and found to be upregulated in atrophying muscles of tumour bearing, starved, or denervated mice. Importantly, its acute inhibition reduced autophagy flux and spared muscle mass in these catabolic conditions [7, 8]. However, prolonged *Mytho* inhibition for months in physiological condition led to the onset of a myopathy, characterized by ER expansion with formation of tubular aggregates, accumulation of amorphous material between contractile proteins, and increase of aberrant mitochondria, which altogether triggered a muscular force drop [7, 8]. Thus, fine-tuning of MYTHO level greatly impacts on muscle function. A knock-out (KO) of this gene in *Caenorhabditis* *elegans (myt-1)* confirmed its role in autophagy and in autophagosome formation. Importantly, *myt-1* inhibition reduced the animal lifespan while its induction improved the health-span of aged worms [6]. However, since *Mytho* is broadly expressed also in different mouse tissues [8], its relevance for development and tissues function remained to be addressed, especially in vertebrates.

Therefore, in this study, we generated a zebrafish *mytho* KO line to dissect Mytho functions. Our results confirmed the involvement of Mytho in the regulation of autophagy and in maintaining tissue integrity.

## Results

### Characterization of *mytho* expression during development and in adult tissues

In zebrafish genome, *mytho* is present as a single-copy gene located on chromosome 18. The encoded protein is highly conserved and shares 88% and 86% identity with humans and mice proteins, respectively.

The *mytho* full-length coding region was sequenced in two fragments (Figs. S1 and S2) and found to contain only five different nucleotides, leading to three aminoacidic differences, compared to the NM_001082813 sequence that has been deposited in the meantime (Fig. S2). Moreover, we found that the presence of a cryptic splicing site in *mytho* exon 7 leads to transcripts that differ for the presence or absence of 33 nucleotides (Figs. S1 and S2). RT-PCR analysis of this region during development and in adult tissues showed that the two expected bands were present in almost all developmental stages and tissues analyzed, suggesting that both splicing sites are used in zebrafish, with the shorter fragment expressed at higher levels (Fig. S3).

**Fig. 1:**
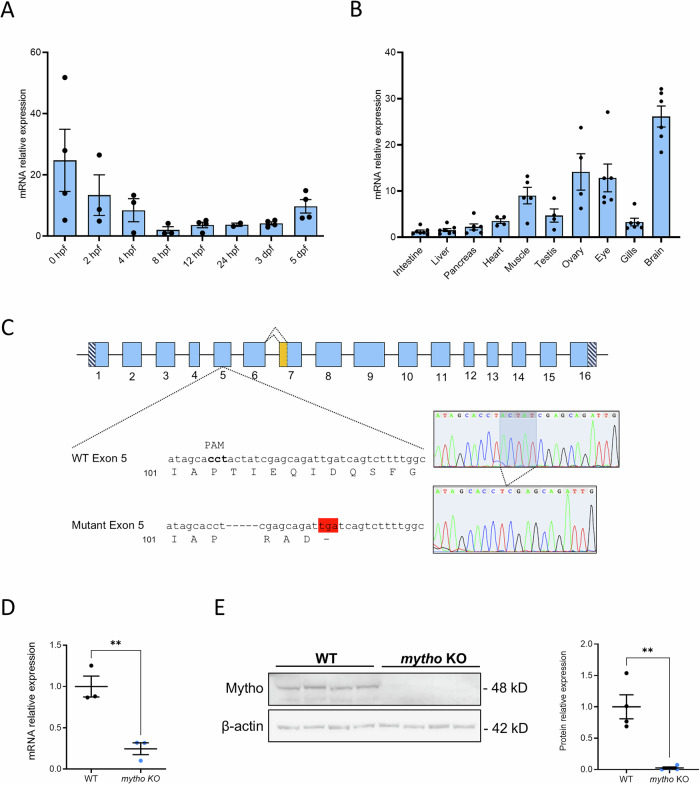
*mytho* expression during development and adulthood and generation of a mytho KO zebrafish line. **A** Analysis by RT-qPCR of the relative abundance of *mytho* transcripts in whole zebrafish embryos and larvae, from 0 hpf to 5 dpf. The reference sample is 8 hpf-time point. The gene *ube2a* was used as housekeeping gene. The bar graph shows the mean ± SEM. Data were generated from 3–4 replicates of samples, each comprising 15 wild-tye (WT) embryos or larvae at the same developmental stage. **B** Analysis by RT-qPCR of the relative abundance of *mytho* transcripts in 6-mpf zebrafish tissues. The reference sample is the intestine, and *arp* was used as housekeeping gene. The bar graph shows the mean ± SEM. Data were generated from tissue samples collected from 4-7 WT animals. **C** Schematic representation of the zebrafish *mytho* gene with nucleotide and amino acid sequences of mutated exon 5 showing the 5-bp deletion (represented by dashes) generated by CRISPR/Cas9 approach. Premature stop codon, generated by loss of frameshift, is highlighted in red. The yellow portion on exon 7 represents the 33-nucleotide region that can be present or absent based on the splicing site used. Dotted lines illustrate the splicing alternatives. **D** RT-qPCR of *mytho* expression in 5-dpf WT and *mytho* KO larvae. The gene *arp* was used as housekeeping gene. Data were generated from 3 biological replicates, each comprising 15 WT larvae. Statistical significance was tested with a general linear model (*F*_1,4_ = 26.87, *p* < 0.01). **E** Western blot analysis of Mytho protein in brains of 4-mpf WT and mutants. Beta-actin was used as housekeeping protein. Data were generated from tissue samples collected from 4 animals per genotype. Statistical significance was tested with a general linear model (*F*_1,6_ = 25.8, *p* < 0.01). (**p < 0.01).

*mytho* temporal expression pattern during development revealed high levels of maternal *mytho* transcripts at the one-cell stage, followed by a progressive decline due to clearance of these mRNAs during the maternal-to-embryonic mRNA transition. After that, maternal transcripts were replaced by the corresponding embryonic mRNA and *mytho* expression gradually increased (Fig. 1A).

At 0.5 and 2 h post fertilization (hpf) *mytho* transcripts were well evident and localized in the blastodisc area, as highlighted by whole mount in situ hybridization. Subsequently, the signal declined, becoming undetectable at 8 hpf and turning visible again at 24 hpf. At this stage, the signal was found mainly in the head, while at 5 days post fertilization (dpf) the expression was particularly evident in correspondence of the chondrocranium, swim bladder and intestine, as confirmed by vibratome sections of 5-dpf samples (Fig. S4).

Finally, RT-qPCR analysis revealed that, in zebrafish as in mouse model [6], *mytho* is expressed in many organs of the adult, with the highest expression levels found in brain, gonads, eye and skeletal muscle (Fig. 1B).

### Generation of a stable *mytho* zebrafish mutant line by CRISPR/Cas9

Using a CRISPR/Cas9 approach, we generated a mutant line characterized by a 5-nucleotide deletion in correspondence of exon 5, resulting in a frameshift mutation leading to a premature stop codon (Fig. 1C). The resulting putative truncated protein is 106 amino acids long: the first 103 match the wild-type (WT) Mytho protein and the last three result from the frameshift.

The KO of the gene was confirmed by the statistically significant reduction of *mytho* transcript levels in 5-dpf homozygous mutant larvae compared to WT (Fig. 1D), a result consistent with nonsense-mediated mRNA decay (NMD) due to premature stop codon [9]. Moreover, Western blot analysis of WT and mutant adult brain lysates revealed Mytho protein only in WT samples (Fig. 1E). Despite the presence of two *mytho* transcripts, Western blot analysis revealed only one protein isoform, possibly reflecting the lower expression of the longer transcript and the minimal difference (11 amino acids) between the two translated isoforms (Fig. S3).

### *mytho* KO showed lower resistance to oxidative stress, increased mortality during transition to juvenile stage, abnormal muscle structure and impaired motility

As a first approach to verify whether *mytho* plays a role during zebrafish development, we analyzed morphological traits considered relevant markers of proper larval growth. Body length and eye diameter of WT, heterozygous and homozygous *mytho* mutants at 5 and 16 dpf (Fig. S5A–D) did not show any statistically significant difference between genotypes, suggesting no direct effect of this protein on body development.

**Fig. 2:**
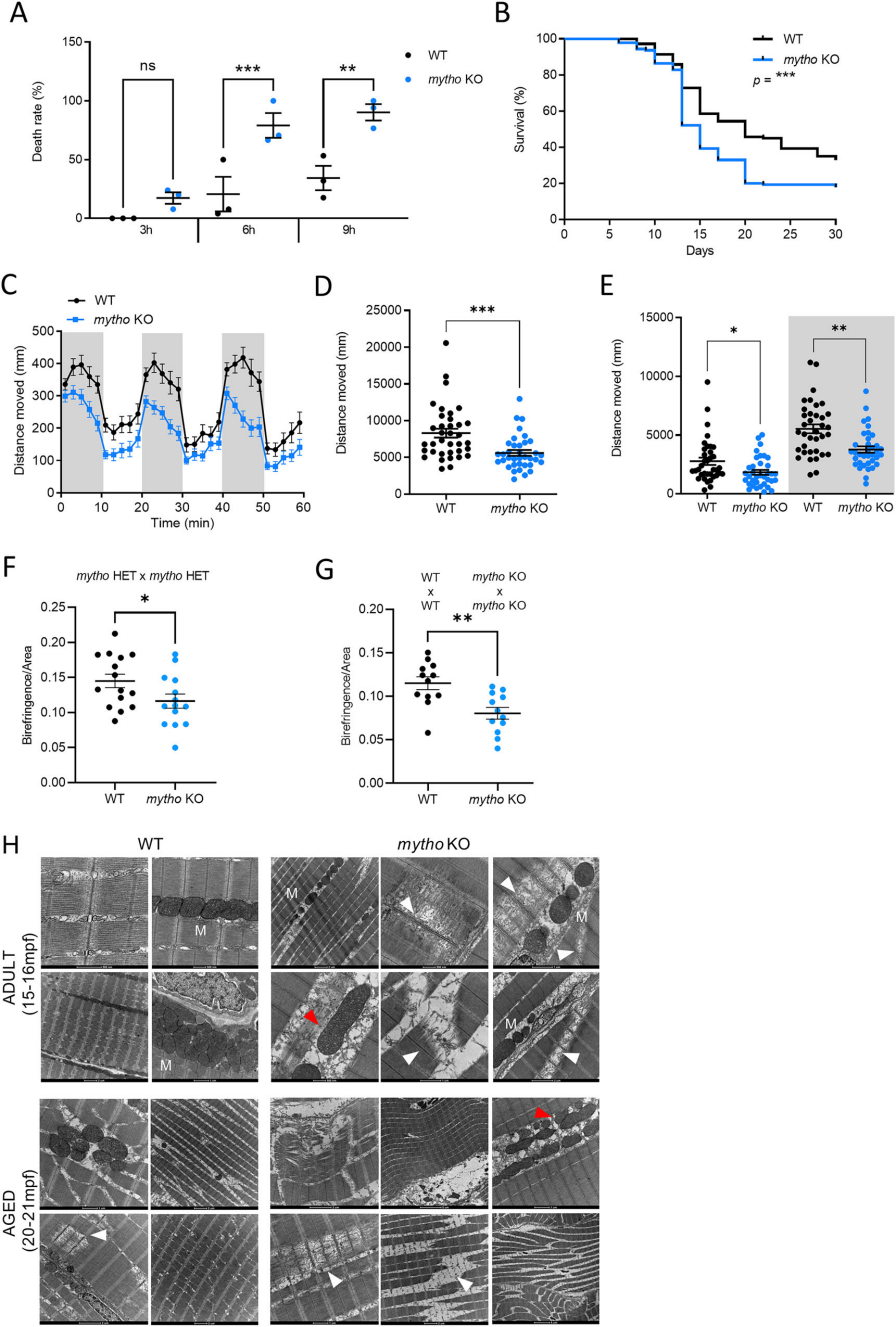
Survival, locomotor behavior and muscle phenotype of *mytho* KO zebrafish. **A** Graph showing WT and *mytho* KO larval resistance to oxidative stress. 5-dpf larvae were exposed to 2 mM hydrogen peroxide and monitored after 3, 6 and 9 h of treatment. Dead animals are expressed as %. Data were generated from 3 biological replicates, for a total of 106 WT and 106 *mytho* KO larvae across replicates. Statistical significance was tested with a beta regression model for the effect of both genotype (*χ*^2^ = 20.362, df=1, *p* < 0.001) and time (*χ*^2^ = 54.2811, df=2, *p* < 0.001) on mortality. P-values in post-hoc pairwise comparisons were adjusted using the Tukey method. **B** Kaplan–Meier survival curves of WT and *mytho* KO zebrafish during the first month of life. Data were generated from three biological replicates, each comprising 50 juveniles per genotype. Statistical significance was determined by Log Rank test. **C**–**E** Locomotor activity analysis of WT and *mytho* KO 5-dpf larvae. Larvae were adapted to light and then stimulated by alternating dark and light cycles. **C** Curves representing the distance moved by WT and *mytho* KO larvae during the dark (grey background) and light phase (white background) in time intervals of 2 min. **D** Total distance moved by WT and *mytho* KO during the entire protocol. Statistical significance was tested with a general linear model (*F*_1,70_ = 16.73, *p* < 0.001). **E** Total distance moved during light (white background) and dark phases (grey background). Data were generated from 3 biological replicates, each comprising 12 larvae per genotype. Graphs show the mean ± SEM. Statistical significance was tested with a linear mixed model for the effect of both genotype (*F*_1,70_ = 15.25, *p* < 0.001) and presence or absence of illumination (*F*_1,70_ = 111.01, *p* < 0.001) on travelled distance. *P*-values in post-hoc pairwise comparisons were adjusted using the Tukey method. Birefringence analysis in larval skeletal muscle of WT and *mytho* KO 5-dpf larvae, obtained by crossing heterozygous animals (**F**) and by crossing animals of the same genotype (WT x WT or *mytho* KO x *mytho* KO) (**G**). In both cases, data were collected from 3 biological replicates from WT (*n* = 15 in graph F and *n* = 12 in graph G) and *mytho* KO larvae (*n* = 14 in F and *n* = 12 in G). Graphs show the mean ± SEM. Statistical significance was tested with a general linear model ((**F**) *F*_1,27_ = 4.27, *p* < 0.05; (**G**) *F*_1,22_ = 12.02, *p* < 0.01). **H** Representative TEM micrographs of adult skeletal muscle from 2 WT (aged 15 mpf and 21 mpf) and 2 *mytho* KO animals (aged 16 mpf and 20 mpf). White arrowheads indicate myofibrillar fragmentation and fiber damages, red arrowheads indicate enlarged mitochondria. (*p < 0.05, **p < 0.01, ***p < 0.001).

To verify *mytho* involvement in stress resistance, we treated WT and *mytho* KO larvae with H_2_O_2_ and checked animal survival at 3 h, 6 h, and 9 h post treatment. Importantly, an increased mortality was revealed in *mytho* KO compared with WT (Fig. 2A) and, as expected, the treatment effect on survival rised over time in both WT and *mytho* KO larvae. Consistently, when we measured larval survival rate during the first month of life, KO larvae showed a significantly higher mortality rate during the larvae-to-juvenile transition, a sensitive period during which activation of the autophagic process is required to overcome the shift between yolk depletion and exogenous feeding [10] (Fig. 2B).

Locomotor behaviour was then analyzed by the “light-dark locomotion test”, in which larvae are exposed to cycles of light-dark switch after 30 min of light acclimation. WT zebrafish larvae showed the canonical movement patterns, with an increase of locomotor activity after the light-to-dark transition and a decrease after the dark-to-light transition. Compared to WT, *mytho* mutants presented a significant reduction in total distance moved (Fig. 2C), with lower locomotor activity during both the dark and light phases (Fig. 2D, E). We then performed a thigmotaxis assay, a behavioural test for analysis of anxiety, adopted in both mammals and zebrafish models [11]. In normal conditions, zebrafish larvae explore their surroundings but show a preference to stay close to the boundary of their container. While increased stress or anxiety levels can accentuate this preference, spending more time in the centre of the arena is a behaviour that suggests reduced anxiety or impaired risk estimation.

Our results revealed no differences in the movement and time spent in the outer zone (i.e. the periphery of the well) between mutants and WT, thus excluding the possibility that the observed locomotion alteration may be linked to anxiety (Fig. S5E, F).

To analyze larval muscular structure, we evaluated the birefringence index of trunk skeletal muscle, a sensitive indicator of muscle size and integrity in translucent zebrafish larvae (Fig. S5G). We observed a statistically significant reduction of the birefringence in *mytho* KO mutants obtained by crossing heterozygous adults (zygotic mutants) (Fig. 2F) and an even stronger birefringence reduction in maternal-zygotic mutants, likely due to the absence of *mytho* maternal transcripts during development (Fig. 2G). Moreover, adult ultrastructural analysis showed that mutant muscle presented a higher rate of myofibrillar fragmentation and destruction together with fibre damage (white arrows) (Fig. 2H). These are considered common features in aged muscles (Fig. 2H, lower panel) and myopathic diseases [12]. In line with that, mitochondria of *mytho* KO were more elongated (red arrows) (Figs. 2H and S5H), recapitulating a typical characteristic of aged muscles due to mitophagy flux impairment [13]. Thus, *mytho* KO zebrafish might develop a marked muscle aging-associate phenotype earlier than WT.

### Autophagy flux was impaired in muscle and brain of *mytho* KO zebrafish

**Fig. 3:**
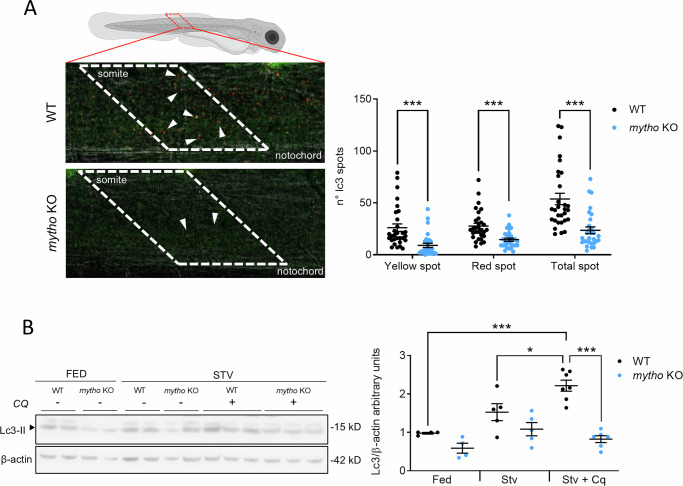
Autophagy flux in muscle and brain of mytho KO larvae. **A** Schematic representation of somite’s region selected to monitor autophagic flux in 5-dpf WT and *mytho KO* zebrafish larvae, after injection at the one-cell stage with the mCherry-GFP-LC3 DNA plasmid (yellow puncta identify autophagosomes and red puncta evidence autolysosomes). Data were generated from three biological replicates, for a total of 30 WT and 29 *mytho* KO larvae across replicates. Graphs show the mean ± SEM. Statistical significance was tested with generalized models assuming Poisson error distribution ((yellow) *χ*^2^ = 30.655, df = 1, *p* < 0.001; (red) χ^2^ = 22.517, df=1, *p* < 0.001; (total) χ^2^ = 33.004, df=1, *p* < 0.001). **B** Western blot analysis of Mytho protein in brains of 4-mpf regularly fed (FED) or starved (STV) WT and mutants, treated with or without chloroquine (CQ) to block the autophagic flux. Both Lc3 forms were detected and the lipidated Lc3-II was normalized by ß-actin, used as housekeeping protein for quantification. Data were generated from whole brain tissue collected at two different time points from 16 WT (4 FED, 5 STV and 7 STV + CQ) and 15 KO (4 FED, 5 STV, 6 STV + CQ). Graphs show the mean ± SEM. Statistical significance was tested with a linear model for the effect of genotype (F_1,25_ = 45.93, *p* < 0.001), treatment (F_2,25_ = 12.50, *p* < 0.001) and their interaction (F_2,25_ = 7.73, *p* < 0.01) on autophagic flux. P-values in post-hoc pairwise comparisons were adjusted using the Tukey method. (*p < 0.05, ***p < 0.001).

To investigate Mytho involvement in autophagy in the zebrafish model, as already demonstrated in mammalian cell lines, *C. elegans* and mouse skeletal muscle [6], we injected one-cell stage WT and *mytho* KO embryos with a tandem mCherry-GFP-LC3 reporter to monitor the autophagic flux. With this approach, yellow puncta visualize the autophagosomes, as LC3 integrated into autophagosomes’ membrane maintains both red and green fluorescence. In contrast, red puncta depict the autolysosomes due to GFP quenching under acidic lysosomal conditions and the stability of the mCherry signal. We observed a reduction of both autophagosomes and autolysosomes in skeletal muscle of 5-dpf mutants (Fig. 3A), suggesting an impairment in the autophagosome formation and a decreased autophagic flux in skeletal muscle.

We then analyzed the autophagic flux in zebrafish brain, the organ with the highest *mytho* transcripts expression, by starving adults of both sexes, treating them with chloroquine, a well-known autophagic flux blocker, and comparing them with fed individuals. Western blot analysis of Lc3-II in adult brains revealed a different response to treatment across genotypes, with a lower accumulation of this protein after starvation and chloroquine treatment in *mytho* mutants. This result suggests a reduced autophagic flux also in this organ (Fig. 3B).

### *mytho* deletion caused organs degeneration and increased onset of testicular seminomas

**Fig. 4:**
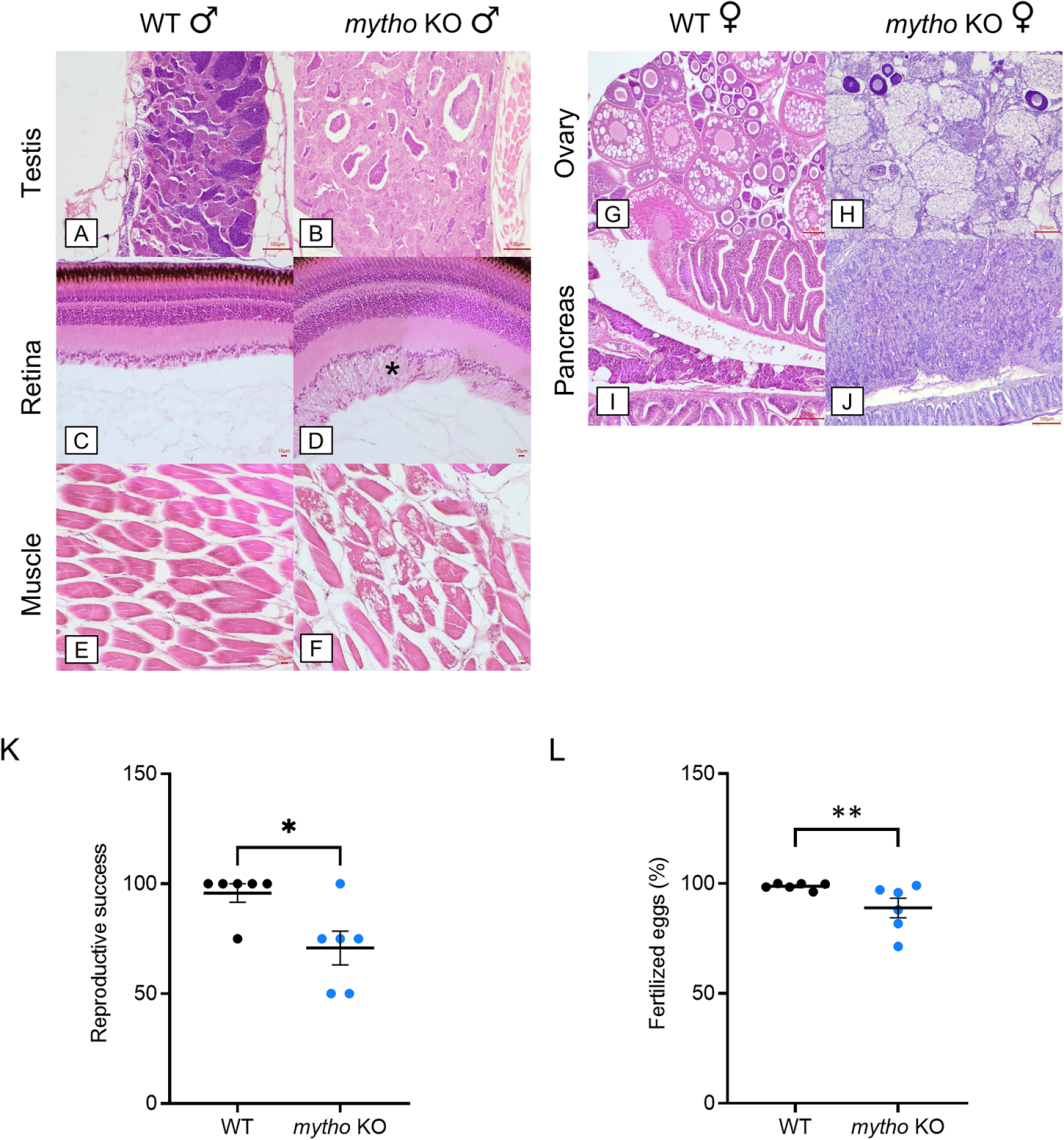
Comparative morphological analysis of adult WT and *mytho* KO and evaluation of reproductive capability. Left panel: comparison between male WT and *mytho* KO anatomical features of testes (**A**, **B**) retinas (**C**, **D**) and skeletal muscles (**E**, **F**). Right panel: comparison between female WT and *mytho* KO anatomical features of ovaries (**G**, **H**) and exocrine pancreas (**I**, **J**). Scalebar is reported in each figure. The number of animals used for histological analyses is reported in Table S1. **K** Graph showing the percentage of reproductive success of WT and *mytho* KO males. Data were generated from 6 WT and 6 *mytho* KO males (aged 8-10 mpf), each mated 4 times with different age-matched WT females to exclude specific male-female interactions. Graphs show the mean ± SEM. Statistical significance was tested with a beta regression model (*χ*^2^ = 6.104, df = 1, *p* < 0.05). **L** Percentage of fertilized eggs over the number of non-coagulated eggs calculated for each successful mating event. Graph shows the mean ± SEM. Statistical significance was tested with a beta regression model (χ^2^ = 7.808, df = 1, *p* < 0.01). (*p < 0.05, **p < 0.01).

Four organs were compared histologically in WT and *mytho* KO adult zebrafish at different ages: gonads (testes and ovaries), exocrine pancreas, retina and skeletal muscle (Table S1). The principal lesions found throughout the different groups were mostly located in both male and female gonads (Fig. 4B, H), with a higher incidence in KO groups at any age and mainly sparing WT groups (Fig. 4A, G). The striking findings in KO males accounted for testicular neoplasia (seminomas) (Figs. 4B and S6), observed in 5 out of 12 subjects, and testicular degeneration in one male (Table S1). Four of the testicular seminomas were characterized by a “classic pattern” consisting of moderate to high cellular proliferation of poorly demarcated, not encapsulated germ cells at different developmental stages. The overall histological architecture of the testes was obscured by sheaths and small clusters of round to polygonal spermatogonia with abundant and weakly eosinophilic to amphiphilic cytoplasm, a round to oval central nucleus with clumped to vesicular chromatin and one evident nucleolus. Atypical features were overall mild to moderate with occasional evidence of mitotic activity. Multifocal spermatocytes and spermatids were also evident in small aggregates. One seminoma was characterized by a cystic pattern with large areas of ectasia of neoplastic seminiferous tubules and reduced spermatogenesis. No testicular neoplasia was observed in WT males and only 2 out of 11 samples were associated with a diffuse moderate hyperplasia of the spermatogonia lineage. The main changes observed in the female gonads were spread in both genotypes with a higher incidence in *mytho* KO females (9 out of 9 subjects) when compared to the WT group (5 out of 6 subjects). Ovarian degeneration was the main pathological change observed in both groups. However, while in the WT females the ovarian degeneration was quite localized and limited (Table S1), in the *mytho* KO ones the degenerative changes were extended and associated to egg retention, likely favouring the occurrence of oophoritis and coelomitis as a consequence of yolk protein leakage within the coelomic cavity, triggering a foreign body reaction [14]. Regarding the pancreatic tissue, both male and female WT subjects did not show any morphological change (Fig. 4I), whereas the KO fish exhibited exocrine pancreas acinar degeneration with intracytoplasmic vacuoles in one male and 2 females and a primary pancreatic neoplasia morphologically consistent with pancreatic carcinoma in one female (Fig. 4J). The main pathological changes in the retina consisted of degeneration with vacuolation at the level of the ganglion cell layer. This change was observed only in one WT male with mild severity, whereas remaining WT subjects did not show retinal morphological changes (Fig. 4C). Retinal degeneration was instead present in 10 *mytho* KO fish (6 males and 4 females) (Fig. 4D). Similarly, the skeletal myofibers were normal in WT subjects of both sexes (Fig. 4E), while they were characterized by degeneration and occasional single cell necrosis in *mytho* KO animals (2 males and 4 females), with the main morphological changes dominated by cell swelling, hypereosinophilia, vacuolation, loss of striation, fragmentation, and rupture of myofibers (Fig. 4F).

Since in mutant ovaries the depicted morphological alterations were commonly observed also in WT, although with a milder phenotype, we chose to focus our study specifically on males’ gonads due to the more notable morphological abnormalities, including the presence of seminomas, which can suggest a potential impairment of male reproductive function. To study reproduction capacity, we crossed WT and mutant males with WT females. Reproductive success, represented by the percentage of successful breeding trials, was significantly reduced in male *mytho* KO (Fig. 4K). Coherently, the percentage of fertilized eggs in the successful breeding events was also reduced (Fig. 4L).

### *mytho* inhibition caused ultrastructural alterations in testis

**Fig. 5:**
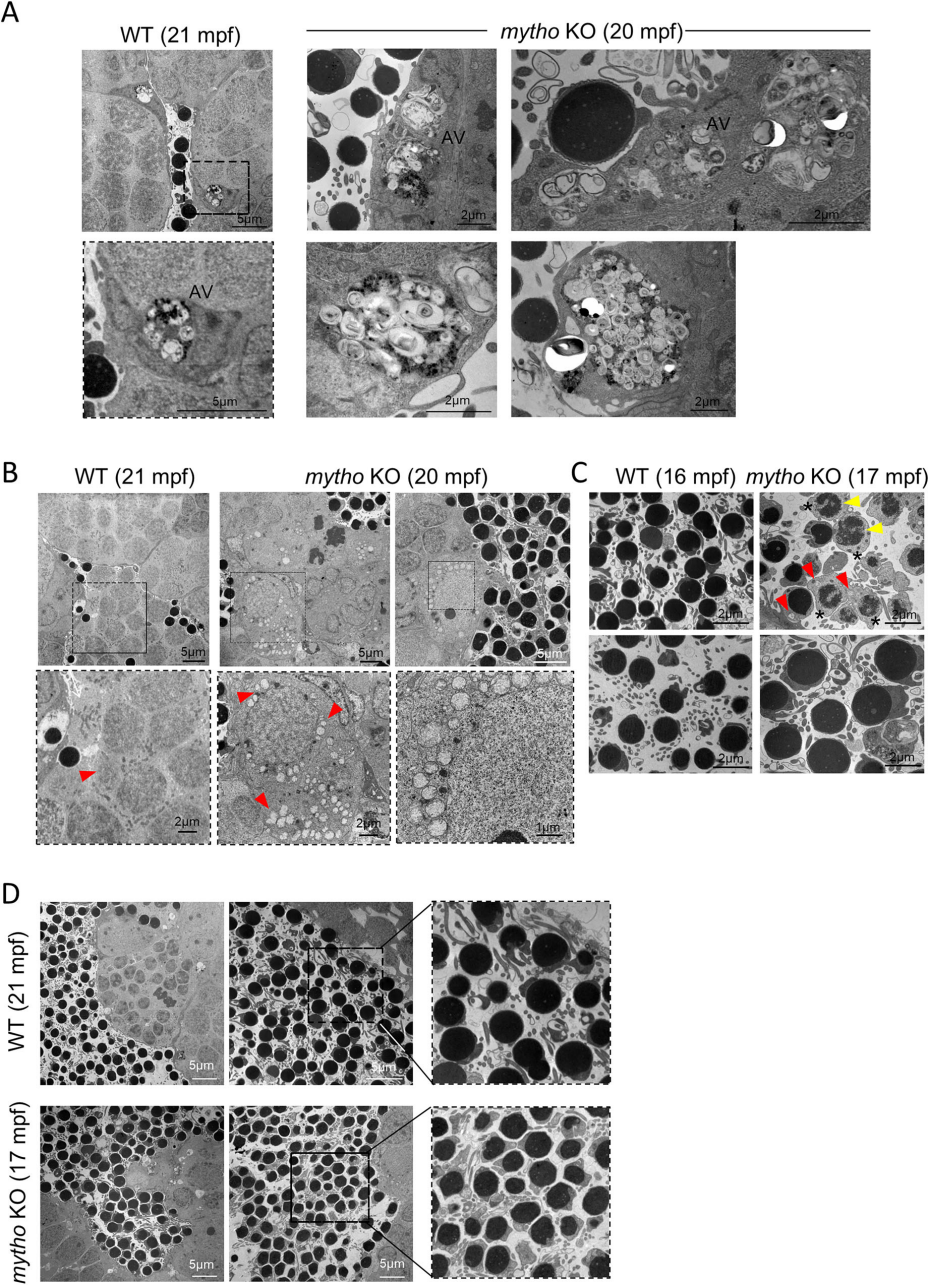
Ultrastructural analysis of testicular alterations in *mytho* KO zebrafish. **A** Representative TEM micrographs from 4 WT (1 aged 16 mpf, 1 aged 17 mpf and 2 aged 21 mpf) and 4 *mytho* KO (2 aged 17 mpf and 2 aged 20 mpf) testes showing a higher presence and size of autophagic vesicles (AV) with material accumulation, residual membranes and undegraded organelles. Scalebar is reported in each figure. **B** Representative TEM micrographs of adult WT and *mytho* KO testes showing the presence of swollen mitochondria devoid of cristae (red arrowheads). Scalebar is reported in each figure. **C** Representative TEM micrographs of testis showing immature sperm with remaining of cytoplasm (asterisks), swollen mitochondria devoid of cristae (red arrowheads) and uncondensed nuclear material (yellow arrowheads) in *mytho* KO samples. Scalebar is reported within each figure. **D** Representative TEM micrographs of testis from adult WT and *mytho* KO animals evidencing differences in sperm nuclei circularity.

Electron microscopy analysis of KO testes revealed that the cytoplasm of Sertoli cells was filled by very large autophagic vacuoles, containing membrane remnants and electron dense material (Fig. 5A), confirming an impairment of autophagy-lysosomal system. Although these vacuoles were also present in the WT (Fig. 5A, left panel), since it is known that autophagy is active in Sertoli cells [15], they were rare and definitely smaller in size, compared to *mytho* KO. Moreover, in agreement with the abnormal mitochondria morphology of *mytho* KO muscle cells [6], both germ and somatic cells of mutant testes frequently showed swollen mitochondria associated to loss of cristae (Fig. 5B, red arrowheads), whereas in the WT testes mitochondria appeared to be more electron-dense (Fig. 5B).

Additionally, images of seminiferous tubules’ lumen revealed the presence of a great number of immature or altered spermatids mixed with spermatozoa (Fig. 5C). These spermatids have altered mitochondria (red arrowheads) and some of them also show uncondensed nuclear material (yellow arrowheads). Furthermore, an extensive number of sperm still contained superfluous cytoplasmic material, that in physiologic condition is normally condensed and discarded as “residual bodies” during spermiogenesis (asterisks).

In addition, we observed subtle differences in nuclear morphology between WT and KO spermatozoa, with the nuclei of mutants displaying a less circular shape in some regions (Fig. 5D). Finally, one of the four mutant testes, analyzed by TEM, showed the presence of massive undegraded material vesicles and deformed nuclei, confirming the occurrence of a pathological condition (Figure S7).

### Transcriptomic signatures revealed an altered DNA damage response

To get insights of the molecular mechanisms that induce spermatogenesis abnormality and triggers testicular tumour development, we performed transcriptome analysis by RNA sequencing (RNA-seq) of zebrafish WT and KO testes. During organ dissection, two of the KO testes were classified as seminoma-bearing testes, based on their remarkable size and rounded shape. This was confirmed both by histological analysis of a tissue’ fragment and by the principal component analysis (PCA). Indeed, while the four WT and the other two KO samples were close although separated, seminoma samples differentially segregated from both (Fig. S8A).

Differential gene expression (DGE) analysis was performed by comparing WT with either KO or seminoma-bearing KO samples. In the KO vs. WT comparison, 896 differentially expressed genes (DEGs) were identified, with 446 upregulated and 450 downregulated in WT (Fig. S8B). The seminoma vs. WT comparison showed 2787 DEGs, of which 1703 were upregulated and the remaining 1084 downregulated in WT (Fig. S8C). When all the KO samples, including seminomas, were compared to WT, a progressive shift of the transcriptome profile was detected. Indeed, the two KO samples showed an expression pattern that was between WT and seminomas (Fig. S8D).

**Fig. 6:**
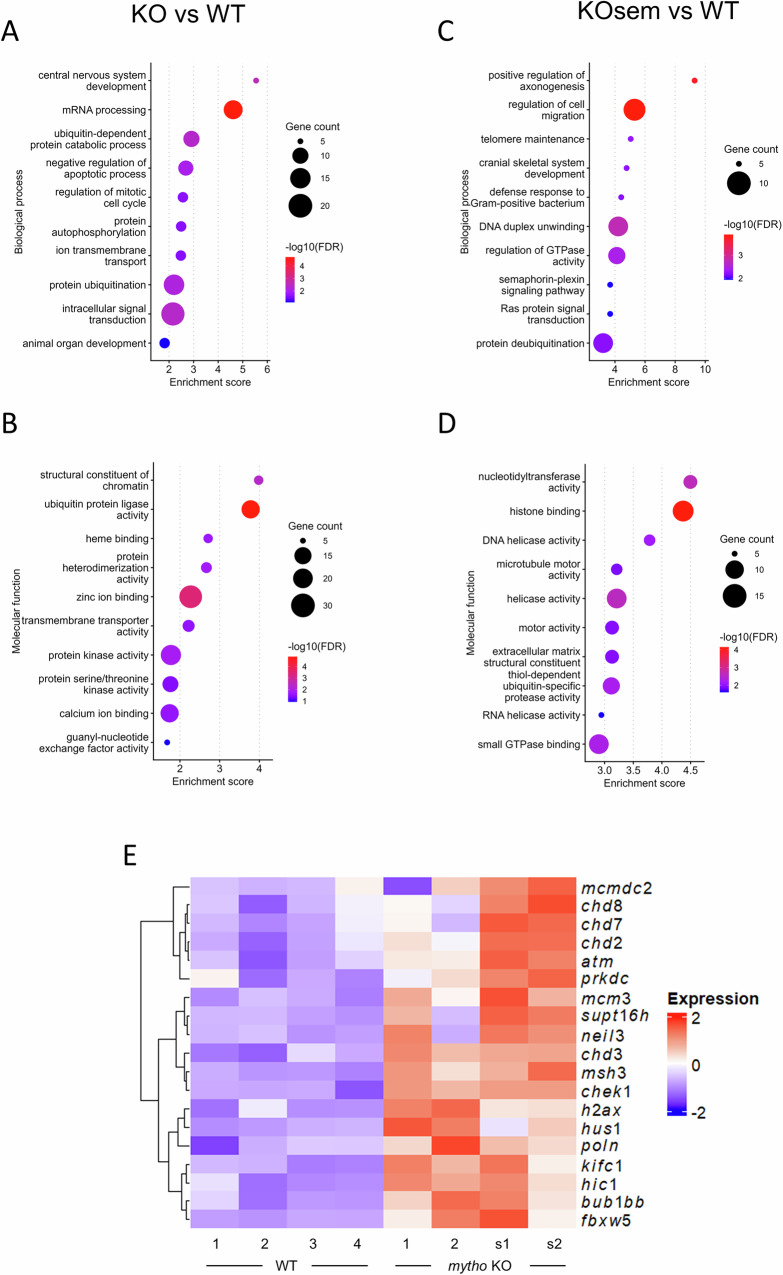
RNAseq analysis of WT and *mytho* KO testes. RNAseq was performed on testes from four 15-mpf WT and four 15-mpf *mytho* KO animals, two of which had developed a seminoma. Bubble plots representing gene ontology enrichment analyses (GOEAs) of the WT vs *mytho* KO (**A**, **B**) and WT vs seminoma-bearing *mytho* KO (**C**, **D**) comparisons. Biological Process and Molecular Function categories were considered. Only GOs represented by 5 or more genes were used for the analyses and the first 10 GOs in term of Enrichment Score (reported in abscissa) are shown in the graph. Size and colour of the bubbles represent respectively the number of genes in each GO and the statistical significance of the enrichment. **E** Heatmap showing expression patterns (z-scaled raw counts) across the samples of DEGs related to genome stability maintenance upregulated in *mytho* KO testes. The genes were manually selected, only keeping the ones whose function has been verified experimentally or with a clear homology to other known genes. Columns correspond to samples, and rows to DEGs. Changes in expression levels are displayed from blue (less expressed) to red (more expressed). Hierarchical clustering of the genes was established using Pearson correlation.

Gene ontology enrichment analysis (GOEA) showed that pathways related to mRNA processing, apoptosis, chromatin structure and regulation of cell cycle were altered in KO animals (Fig. 6A, B). Telomere maintenance, DNA duplex unwinding, histone binding and DNA helicase activity were overrepresented among the upregulated genes in seminoma-bearing KO with respect to WT testes (Fig. 6C, D. For the full GOEA results see Fig. S8–2). In agreement with the presence of many deregulated pathways linked to chromatin structure/accessibility and response to genome instability, genes involved in DNA damage and repair such as *atm*, *h2ax* and *chek1* were upregulated in *mytho* KO testes and kept higher in seminoma-bearing KO, as pointed out by the heatmap displaying some of the genes related to these pathways (Fig. 6E. For the heatmap displaying the downregulated genes see Fig. S8–1).

### *mytho* inhibition enhanced double strand break DNA damage

**Fig. 7:**
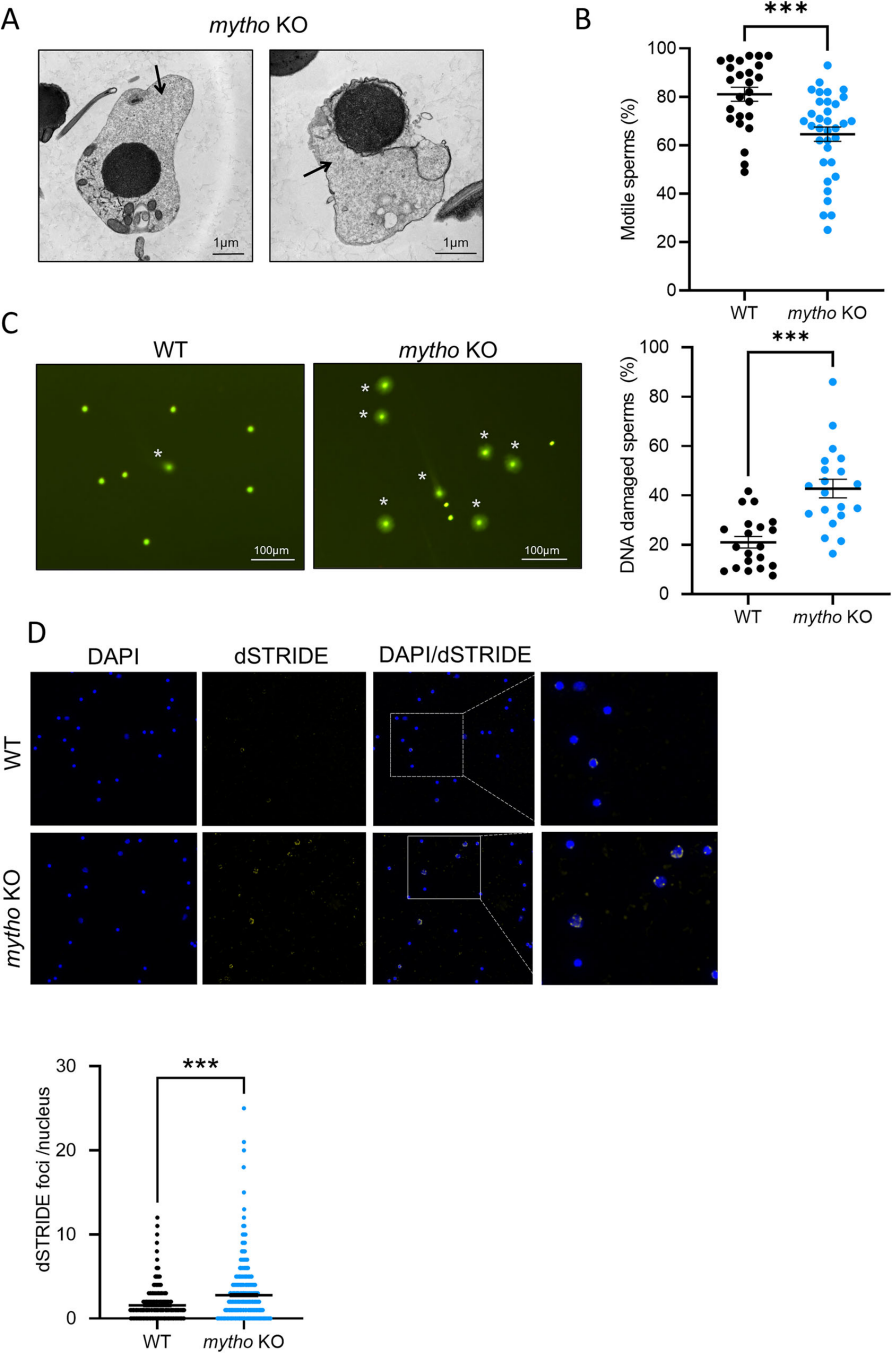
Analysis of sperm quality parameters. **A** Representative TEM micrographs of *mytho* KO immature sperm with still present cytoplasmic material (black arrows). Scalebar: 1 µm. **B** Percentage of motile sperm measured with CEROS Sperm Tracker (Hamilton Thorne Research) in ejaculate collected from 17 WT and 18 *mytho* KO adult zebrafish of 7-14 mpf. Graphs show the mean ± SEM. Statistical significance was tested with a beta regression model (χ^2^ = 17.902, df = 1, *p* < 0.001). **C** Representative images from Halomax assay where DNA-fragmented sperm shows a big, diffused and faint halo (asterisks), while sperm with intact DNA appears as small, compact and bright puncta. Scalebar: 100 µm. Percentage of DNA-fragmented sperm was calculated in ejaculates collected from 20 WT and 20 *mytho* KO adult zebrafish of 7–18 mpf. Graphs show the mean ± SEM. Statistical significance was tested with a beta regression model (χ^2^ = 23.914, df = 1, p < 0.001). **D** Representative images from dSTRIDE staining (IntoDNA) of sperm collected from 2 WT and 2 *mytho* KO adult zebrafish aged 11 mpf. The graph shows the mean ± SEM of double-strand breaks foci number per sperm nucleus. Statistical significance was tested with a generalized linear model with Poisson error distribution (χ^2^ = 93.788, df = 1, *p* < 0.001). (***p < 0.001).

To support the hypothesis of chromatin vulnerability when *mytho* gene is inhibited and identify the cause of testis tissue degeneration and seminoma onset, sperm samples were collected from WT and mutant individuals and analyzed at the ultrastructural level. The *mytho* KO individuals showed a higher percentage of sperm retaining abundant cytoplasm (9.1% of sperm cells *versus* 1.8% in WT), suggesting a maturation delay or an impairment of the spermiogenesis process (Fig. 7A). Next, we analyzed several parameters related to sperm quality without finding differences between genotypes in sperm concentration, viability, velocity and linearity of trajectory (Fig. S9, and Tables S2, S3). Conversely, the percentage of motile sperm was significantly reduced in *mytho* KO (Fig. 7B).

Finally, since the transcriptomic signature suggested an enhanced chromatin vulnerability/instability and DNA damage in *mytho* deficient zebrafish, we analyzed DNA fragmentation in *mytho* KO sperm, using a test based on sperm chromatin dispersion (SCD). Sperm of KO males showed an increased number of nuclei with blurred halo, indicating increased DNA fragmentation compared to WT (Fig. 7C).

To determine the type of DNA damage found in sperm, we performed DNA damage analysis by using dsSTRIDE, which recognizes DNA double strand breaks, on spermatozoa obtained from 11-month-old WT and *mytho* KO zebrafish. We found that ablation of *mytho* increased the number of dsSTRIDE foci, confirming that *mytho* absence promoted double-strand breaks (Fig. 7D) which, in turn, could explain the premature cellular senescence and the onset of cancer.

## Discussion

Autophagy is a highly conserved system which removes misfolded proteins and damaged organelles preserving cellular homeostasis, integrity and function. Although autophagy has been extendedly studied during last decades, there are still many gaps in our understanding of its regulation and pathophysiological relevance.

We have recently discovered a novel gene, *mytho*, that is upregulated under stress conditions by FoxOs and contributes to the maintenance of muscle integrity and healthy ageing in basal conditions [6, 8]. In this work, we aimed to understand how the lack of this protein can affect tissue development and homeostasis in a vertebrate model, *Danio rerio*, thanks to its highly conserved Mytho amino acid identity (87,7%) with humans. Therefore, identifying which tissues are affected by *mytho* KO in zebrafish, will potentially be relevant also for human physiology.

First, this study confirms the role of *mytho/phaf1* in autophagy. Consistently with ours and others published findings [6–8, 16], an impairment in the autophagosome formation and a decreased autophagic flux were detected in skeletal muscle and brain of KO animals. In addition, similarly to other zebrafish mutants or morphants of the autophagic process, e.g. *atg5*, *atg7* and *beclin1* [10, 17], *mytho* mutants showed a lower survival rate during the larval-to-juvenile transition, a stage where autophagy is likely required for managing metabolic changes and supplying energy demands after the resorption of the yolk sac. Locomotion was also reduced in zebrafish *mytho* KO model, in agreement with our previous study in *myt-1* mutant *C. elegans* in which autophagy and motility were both impaired [6].

Moreover, this study highlights that Mytho regulation of autophagy is not limited to the skeletal muscle, but it takes place also in the brain, an organ where *mytho* is highly expressed both in zebrafish and mouse. Finally, the accumulation of undegraded material observed by TEM in testis cells, involving both germ and somatic ones, strongly highlights the occurrence of an autophagic impairment in male gonads and suggests a role for Mytho protein in the regulation of this pathway.

Consistently, we found that reproductive capabilities were significantly reduced in male *mytho* mutants, in agreement with the functional roles of autophagy in the spermatogenetic process [4], as highlighted also by the recent analyses of different zebrafish and mouse mutant lines for autophagic proteins [2, 3, 18–20] or autophagy-regulating genes [21].

In addition to the essential cleaning function, autophagy appears to be an important mechanism in cell differentiation and development, allowing cells to modify their cytoplasmic content and morphology. The crucial role of autophagy for spermiogenesis and male reproduction has already been highlighted [5, 22, 23]. Many autophagy-related genes are actively expressed in human spermatogonia (ATG5, EPG5, TBC1D20) and spermatocytes (ATG7, SIRT1, PRKACA) [24]. The analysis with a web-based application that allows to explore single-cell datasets of zebrafish testis (https://github.com/asposato/zebrafish_testis_fertility) [25], shows that *mytho* (*si:dkey-102f14.5*) expression is high in spermatogonia and spermatocytes, and then lowers in spermatids and spermatozoa, suggesting a possible predominant role in the maturation step of these cells (Fig. S10A). A similar result is obtained analyzing *MYTHO* expression (*C16ORF70*) in a single-cell transcriptomics map of human testis (Fig. S10B) [26]. Moreover, the analysis of aged zebrafish testis revealed that this transcript remains present in germ cells, but its expression increases also in the immune cells, in agreement with the higher abundance of these cells during aging [27].

Autophagy promotes the removal of unnecessary cytoplasm and organelles during spermiogenesis [28], allowing a correct cell differentiation and spermatozoa motility. In humans, cytoplasmic remnants are associated with spermiogenesis arrest and interruption of cytoplasmic extrusion, triggering oxidative stress, peroxidative damage to sperm membrane, and DNA damage [29], finally leading to infertility. In agreement with this, a depletion of the autophagic gene ATG7 in mouse germ cells has been shown to determine a defect in spermatozoa cytoplasmic material removal, with consequent reduction of sperm motility and fertility [19]. Interestingly, we observed similar phenotype, with residuals of cytoplasmic material in spermatids and spermatozoa of zebrafish *mytho* KO. Normally, this material, together with germ cells that undergo apoptosis, is phagocytosed and recycled by Sertoli cells and is rarely found in tubules [30].

Sperm motility impairment in ATG7 KO mouse [19] was associated with the disorganization of the “9 + 2” structure of the flagella and other cytoskeletal components in spermatozoa. Notably, BCAS3 has been associated with cytoskeletal proteins facilitating cell migration and angiogenesis [31] and MYTHO protein has been found closely interacting with BCAS3 [6, 16] in human cells. However, whether sperm motility impairment, observed in our model, is uniquely related to autophagy or through a mechanism dependent on cytoskeletal organization remains to be investigated in future studies.

This work has also demonstrated that *mytho* deletion increased DNA damage, an issue already negatively correlated to sperm quality and performance and, thus, to sperm senescence and lower fertilizing ability [32]. Importantly, the double-strand break fragmentation was enhanced by *mytho* inhibition, possibly explaining the reduction of fertility, the increased number of seminomas in mutant testis and the presence of a pancreatic cancer in one female. This finding suggests a role of Mytho in DNA stability/repair.

The main pathological changes found in mutant zebrafish affected the reproductive system, highlighting a pivotal role for Mytho in the homeostasis of these organs and in the development of neoplasia. Neoplastic lesions were mainly observed in males, with seminoma being the only tumour diagnosed in males KO samples. In zebrafish males, seminoma represents the most frequent naturally occurring neoplasia [33]. Its incidence was found lower than 2% in a histological study of nearly 10,000 2-year-old zebrafish [34], but increased to 17% in WT zebrafish of 30–34 months of age [35], suggesting a physiological association between its onset and zebrafish aging process. A similar increase of seminoma was also found with the silencing of *ambra1a* and *ambra1b* [21], two paralogous genes involved in the positive regulation of autophagy [36].

The pathological changes observed in reproductive traits in females were confined to the ovary. Although degeneration of ovaries and chronic ovarian and coelomic inflammation, associated with degenerating eggs, is a common condition observed in zebrafish facilities and referred to as Egg-Associated-Inflammation (EAI), the higher incidence and severity of these lesions found in mutant subjects suggests a role for Mytho in the development of this syndrome, whose pathogenesis is still unclear [14]. Finally, the muscle lesions, the pancreatic and retinal degeneration we described in adult mutant zebrafish are mainly identifiable in aged individuals, reinforcing the hypothesis of a crucial role of Mytho in the aging process.

In conclusion, this study confirms the conserved role of Mytho in autophagy and in maintenance of DNA stability, establishing this protein as a key regulator of cell homeostasis and tissue function in *D. rerio* model. Intriguingly, the link between *mytho* ablation and increased susceptibility to DNA damage, suggests a potential role for Mytho in protecting cells from cancer onset.

## Methods

### Animal maintenance and handling

Zebrafish (*D. rerio*) larvae and adults were maintained in the facility of the University of Padova, at a constant temperature of 28.5 °C under a 12:12 light:dark (LD) cycle, according to standard procedures. WT lines used in this work included Tuebingen, Giotto and Umbria strains. Embryos were obtained from natural spawning and kept in artificial Fish water. For zebrafish anaesthesia or euthanasia, tricaine (E10521, Sigma-Aldrich, Milan, Italy) was added to fish water at 0.16 mg/mL or 0.3 mg/mL, respectively. Husbandry and experimental procedures were carried out in accordance with the Italian and European Legislation for the Protection of Animals used for Scientific Purposes (Directive 2010/63/ EU) and were previously approved by the Animal Ethics Committee of the University of Padua and by the Italian Ministry of Health (Authorization Number 568/2016-PR). Experimental sample size was minimized in accordance with ethical guidelines while ensuring adequate statistical power.

### Generation and genotyping of the zebrafish *mytho* KO mutant lines

The zebrafish mutant line *mytho* KO (recorded in ZFIN as ia39) was generated by CRISPR/Cas9-mediated genome editing. A single guide RNA (sgRNA) (TGATCAATCTGCTCGATAGT-AGG) was designed to specifically target a CRISPR site within exon 5 of the *mytho/phaf1* gene (initially identified as sequence si:dkey-102f14.5-203, now updated to sequence NM_001082813). This sgRNA was designed using the Breaking-Cas online tool (https://bioinfogp.cnb.csic.es/tools/breakingcas/index.php?gset=SARS2), which utilizes algorithms to identify exon sites with high efficacy and minimal off-target effects, to specifically target an optimal CRISPR sequence.

The DNA oligonucleotide targeting *mytho/phaf1* and the constant oligonucleotide are reported in Table S4. gRNA synthesis templates were generated following the protocol by [37]. Briefly, single-stranded DNA (ssDNA) oligonucleotides were annealed and filled in with T4 DNA polymerase. The resulting template was transcribed in vitro using the SP6 transcription kit (MEGAscript SP6 kit, AM1330, ThermoFisher Scientific, Monza, Italy), adhering to the manufacturer’s guidelines. At the 1-cell stage, embryos were injected with 1 nl of a solution containing 280 ng/μL of Cas9 protein (M0646, New England Biolabs, Milan, Italy) and 100 ng/μL of sgRNA, with 0.05% phenol red as an injection tracer. Genomic DNA extracted from 5-dpf F0 embryos was analyzed to evaluate gRNA activity in producing DNA indel mutation by a heteroduplex mobility assay (HMA). Putative F0 founders were raised to adulthood and mated with WT zebrafish. Their embryos were genotyped to confirm germline transmission and identify the desired frameshift mutation in the F1 generation. Heterozygous F1 mutants carrying the selected mutation were bred with WT fish to produce heterozygous F2 offspring. These F2 heterozygotes were then crossed to obtain homozygous mutant embryos in the F3 generation. Heterozygotes and homozygotes were identified at early stages through DNA isolation, PCR amplification of the mutated region using primers manually designed, and sequencing of the PCR amplification. Genomic DNA was extracted using the HotSHOT protocol. Primer sequences are provided in Table S5.

### RNA isolation and RT-qPCR assay

Total RNA was extracted either from pools of embryos or larvae, or from single organs, using TRIzol reagent (15596026, ThermoFisher Scientific) and complementary DNA was generated using 1 µg of total RNA using the SuperScript IV reverse transcriptase (18090050, ThermoFisher Scientific) and random primers, following the manufacturer’s instructions.

Real-time PCR detection of mRNA expression was performed on QuantStudio 5 Real-Time PCR Systems (ThermoFisher Scientific) using the Power-UP SYBR®Green PCR Master Mix (A25741, ThermoFisher Scientific). Cycle threshold (CT) values were obtained for each target gene. Fold change values were calculated using the Delta Delta Ct (ΔΔCt) method. All RT-qPCR primers were manually designed to span one intron. Primer sequences are reported in Table S5.

For the RNAseq analysis, the sample preparation followed the procedure described above. RNA was extracted from testes of 4 WT and 4 KO animals of about 15 months of age. After quantification, samples were diluted to a concentration of 100 µg/ml and shipped to Sequentia biotech (Barcelona, Spain) for the analysis.

### RNA sequencing

RNA quality was assessed using an Agilent 2200 TapeStation System (Agilent Technologies). Sample RIN (RNA integrity number) values ranged from 9.3 to 9.9. Total RNA samples (1 µg) were used for library preparation (Illumina’s TruSeq Stranded mRNA LT Sample Prep Kit with poly A selection). Sequencing was performed using an Illumina NovaSeq 6000 System (150 length, paired-end) platform (Illumina, Inc., San Diego, CA).

### RNA-seq data analysis

FASTQ files obtained from the sequencer and the quality of the reads were evaluated using FASTQC v0.11.9 (http://www.bioinformatics.babraham.ac.uk/projects/fastqc/). Next, a trimming step was performed to remove adapters and low-quality bases using BBDuk v35.85 [38] with the following parameters: 35 bp minimum length and 25 minimum quality score. High quality reads were mapped against the *D. rerio* genome (Ensembl GRCz11 reference genome and gene annotation, Ensembl release 107) using STAR v2.7.1a [39]. FeatureCounts v 1.5.1 [40] was used to quantify gene expression as raw fragment counts. Raw counts matrices were loaded in R v4.0.3 [41] and filtered using HTSFilter package v1.30.1 [42] to remove the uninformative genes. Filtered raw counts were processed with EdgeR package v3.32.1 [43] to normalize the raw counts using the TMM method and perform differential gene expression analysis to identify upregulated/downregulated genes. Multiple testing correction was performed with the false discovery rate (FDR) method [44] and significance level was set at FDR < 0.05. Gene Ontology (GO) terms enrichment was analyzed performing hypergeometric tests [45] for each individual term/pathway and FDR correction was applied (FDR < 0.05).

### cDNA cloning, digoxigenin probe preparation and whole mount in situ hybridization

To amplify the entire coding region of *mytho*, two pairs of primers (Table S5 and Figure S1) were selected and used with cDNA obtained from 48-hpf embryos.

To synthesize a digoxigenin (DIG)-labelled riboprobe for whole-mount in situ hybridization (WMISH), a cDNA fragment was amplified using the primer Mytho-F2 and Mytho-R2 (Table S5). The product of the PCR was cloned using pGEM® T-easy vector system (A1360, Promega, Milan, Italy). The antisense and sense DIG-labelled probes were synthesized by in vitro transcription with SP6 RNA polymerase supplemented with DIG-UTP (DIUTPS-RO, Sigma Aldrich), following the manufacturer’s instructions, after plasmid linearization with *ApaI* or *SalI* restriction enzymes, respectively.

WMISH was performed following the protocol previously described [36]. After staining, zebrafish embryos and larvae were equilibrated in glycerol 80% and photographed using a LEICA M165 FC stereomicroscope connected to a LEICA DFC7000T.

Representative 5-dpf stained larvae were then gelatin/albumin embedded for vibratome sectioning. Larvae conserved in 80% glycerol were equilibrated in a medium composed by 0.5% gelatine/30% albumin solution in PBS for 15 min. Polymerization was initiated by adding 35 μL of glutaraldehyde 25% to 500 μL of the medium; then larvae were oriented (either for sagittal or transverse sectioning) and polymerization completed by addition of another 35 μL of glutaraldehyde 25%. After 15 min, the samples were completely solidified and then mounted on the vibratome platform. 30-μm thick sections were cut using a LEICA VT 1000S Vibratome, mounted on slides, and photographed.

### Autophagic flux quantification

The autophagic flux was assessed following different approaches for larvae and adults. In zebrafish larvae, we followed the method proposed by Kimura et al*.* [46] using a mCherry-GFP-Lc3 construct that was transferred from the original plasmid into a pCS2+ plasmid, normally used with zebrafish. 250 pg per embryo of this construct were then injected in one-cell stage embryos. At 5 dpf, anaesthetized larvae were mounted in 1% low melting agarose on depression slides for acquisition under a Nikon C2 confocal microscope equipped with NIS Element Software. The same upper emisomite located above the anus was acquired by z-stacks (2 µm step size) for each larva, using a 40X objective. Fiji-ImageJ software was used for image analysis. For each individual, 6 focal planes uniformly spaced were considered and yellow (deriving from the colocalization of the mCherry red and GFP green fluorescence) and red puncta (deriving from the quenching of GFP fluorescence in the autolysosome acidic environment) were manually scored. The number of yellow and red puncta, together with the total number of autophagic vesicles, were compared between WT and *mytho* KO samples. The mCherry-GFP-Lc3 construct used in this work was subcloned from the original vector of Terje Johansen (The Arctic University of Norway) and kindly shared by Sabine Hilfiker (Rutgers - New Jersey Medical School).

In adult zebrafish, we divided 4-mpf individuals in three different groups: a control group fed normally, a group starved for 4 days, and a group starved for 4 days and treated with chloroquine (C6628, Sigma-Aldrich) dissolved in fish water at a final concentration of 1 mM during the last 24 h. At the end of the treatment, zebrafish were euthanized, the organs were extracted, and protein samples prepared as described in the protein extraction and western blotting section.

### Protein extraction and western blotting

Pools of embryos, larvae or organs from adults were used for protein preparation following the published protocol [47]. After quantification, samples were immunoblotted as previously described [6]. We used the following primary antibodies: anti-C16orf70 (Mytho) (ab181987, Abcam, DBA, Milan, Italy), anti-β-Actin (A1978, Sigma-Aldrich), anti-Lc3B (PA1-16930, ThermoFisher Scientific), and secondary antibodies: Goat Anti-Rabbit IgG (H + L)-HRP Conjugate (#1706515, Bio-Rad, Milan, Italy), Goat Anti-Mouse IgG (H + L)-HRP Conjugate (#1706516, Bio-Rad).

### Birefringence assay

Muscle birefringence was used to assess differences in the muscle fibre structure of 5-dpf larvae and performed as previously described [18]. Two independent experiments were performed: one with larvae from a cross between *mytho* heterozygous (HET) parents and the other using larvae from a *mytho* WT *x mytho* WT and a *mytho* KO *x mytho* KO matings.

### Larvae behavioural assay

Behaviour of mutant and WT larvae was analyzed by light dark transition test as previously described [47]. The track files were analyzed using the Ethovision XT software (Noldus Information Technology, Rome, Italy) to assess the distance moved every 2 min. The experiment was repeated 3 times, using 12 larvae per genotype for each biological replicate. For thigmotaxis assay, the protocol of Schnörr and collaborator was followed [11]. Zebrafish larvae were tracked to analyze the time spent in the periphery of the 24-well plates in response to sudden change in illumination: after an acclimatization step of 6 min with the light on, swimming activity of the larvae was recorded for 4 min in the dark.

### Treatment of larvae with H_2_O_2_

The resistance to oxidative stress of WT and mutant zebrafish larvae was assessed following the assay used in [48]. Briefly, 5-dpf larvae were treated by adding hydrogen peroxide (H1009, Sigma-Aldrich) to the fish water at a concentration of 2 mM. Larvae were kept at 28 °C and their mortality was monitored after 3, 6 and 9 h of treatment.

### Reproductive performance

The reproductive performance of six 8-mpf *mytho* KO and six age-matched WT males was assessed by natural matings in spawning tanks under standard aquarium conditions [49]. The reproductive success was assessed in four independent experiments, spaced 7 days apart to allow the generation of new gametes and to obtain a realistic average of the reproductive performance of each individual. Specific male-female interaction effect was excluded by coupling animals with different partners on each trial. To quantify the reproductive success, we recorded the number of times each couple produced eggs across four different trials. For each successful reproduction, we documented the total number of eggs spawned, the number of fertilized eggs over the total number of eggs and the rate of offspring survival.

### Sperm analysis

For the analyses of ejaculate quality, anaesthetized males were stripped to obtain the sperm. A week prior to stripping, males were separated from females by means of a grid allowing visual but not physical contact.

Ejaculate collection, sperm count, viability and velocity, were obtained and analyzed as previously described [3].

Fragmentation of sperm DNA was measured as an index of DNA damage. The assay was performed using a Halomax kit (Halotech, Madrid, Spain), a diagnostic test specifically designed to measure DNA fragmentation, according to the manufacturer instructions. The analysis is based on a protein depletion treatment and the evaluation of chromatin response. DNA-fragmented sperm shows a big, diffused and faint halo, while sperm with intact DNA shows a small, compact and bright halo. The samples were examined under a fluorescence microscope.

### DNA damage quantification through dSTRIDE (SensiTive Recognition of Individual DNA Ends)

As an additional mean to evaluate the level of sperm DNA damage, we chose the dSTRIDE assay provided by intoDNA (Krakow, Poland). Collected sperm were let adhere onto slides previously coated with poly-D-lysine (A3890401, ThermoFisher Scientific), according to manufacturer instruction. Once the sperm adhered to the slides, they were fixed in PFA 4% and shipped to intoDNA. The dSTRIDE methodology was applied according to the protocol previously described [50].

### Histological and electron microscopy analyses

Hematoxylin/eosin histological analysis of WT and mutant fish was performed as previously described [3].

For ultrastructural analysis of adult muscle and testes we used conventional fixation-embedding procedures as previously described [3].

Sperm ultrastructural analysis was performed with samples collected as previously described and fixed with 2.5% glutaraldehyde in 0.1 M sodium cacodylate buffer. Following fixative removal, samples were resuspended in 0.1 M cacodylate buffer for 2 h at 4 °C and then embedded in 3% low melting agarose. Samples were postfixed in 1% osmium tetroxide + 1% potassium ferrocyanide for 1 h at 4 °C, ethanol-dehydrated and then embedded in a mixture of Epoxy Embedding Medium kit (45359, Sigma-Aldrich) epoxy resin and propylene oxide 1:1 overnight. The following procedure was the same described in [3].

### Statistical analysis

Analyses were performed using R Studio v 2023.03.1 + 446. Repeatability within sample for sperm velocity, linearity of sperm trajectory, sperm motility and viability are reported in electronic supplementary Tables S2 and S3. Since sperm velocity parameters (VAP, VSL, VCL) were all highly correlated (all Pearson *r* > 0.96) a combined index of sperm velocity was generated from the 3 velocity variables using a principal component analysis (PCA). The scores of the first component, accounting for 98.54% of variance and with all sperm traits showing similar positive loadings (Table S2), were used for the following analyses on sperm velocity. Continuous dependent variables (gene and protein expression, Daniovision traveled distance, thigmotaxis distance and time, birefringence, autophagic flux, morphometric parameters, mitochondrial length, sperm velocity, sperm count) were analyzed using linear models. Proportional response variables (mortality, reproductive success, fertilization rate, sperm motility, sperm viability and sperm DNA fragmentation) were analyzed using beta regression models. Small counts (n° of autophagosomes and autolysosomes, n° of stride foci) were analyzed with generalized linear models assuming Poisson error distribution and log-link function. Kaplan-Meier survival curves were analyzed with log-rank test. P-values were obtained from F-statistic for linear models and χ^2^-statistic for the other models. In each model, male genetic group was included as a fixed factor (predictor). Other predictors (e.g., time, age, treatment) were also included when testing their combined effects with genetic group. Subject identity was included as a random factor when accounting for repeated measures within subject (mixed models). *P*-values in post-hoc pairwise comparisons were adjusted using the Tukey method. Assumptions of residuals’ normal distribution and variance homoscedasticity in linear models were checked with Shapiro-Wilk’s and Bartlett’s test, respectively, and by inspection of diagnostic plots. A log-transformation or sqrt-transformation was eventually applied to the raw data to improve distribution. Eventual overdispersion in generalized models was addressed by adding an observation-level random effect. Animals allocation to each group was not randomized but based on genetic identity (mutant *vs*. WT individuals). The investigator was blinded to the genetic identity of each sample during outcome assessment.

## Supplementary information

Supplemental Figures and tables

Data sheets and statistical analysis 1

Data sheets and statistical analysis 2

Gene expression data set 1

Gene expression data set 2

Uncropped blots

## Data Availability

The datasets generated and analyzed during the current study are available in NCBI’s Gene Expression Omnibus (Edgar et al., 2002) and are accessible through GEO Series accession number GSE317380 (https://www.ncbi.nlm.nih.gov/geo/query/acc.cgi?acc=GSE317380).
