# Supplementary material for: Mytho/Phaf1 is required to prevent DNA damage and tissue degeneration in Danio rerio
Source: Cell Death Discov. 2026 Apr 17;12:252. doi: 10.1038/s41420-026-03106-x (PMC13212566; doi:10.1038/s41420-026-03106-x)
Supplement: Supplementary file 1 — Supplemental Figures and tables [file 41420_2026_3106_MOESM1_ESM.pdf]

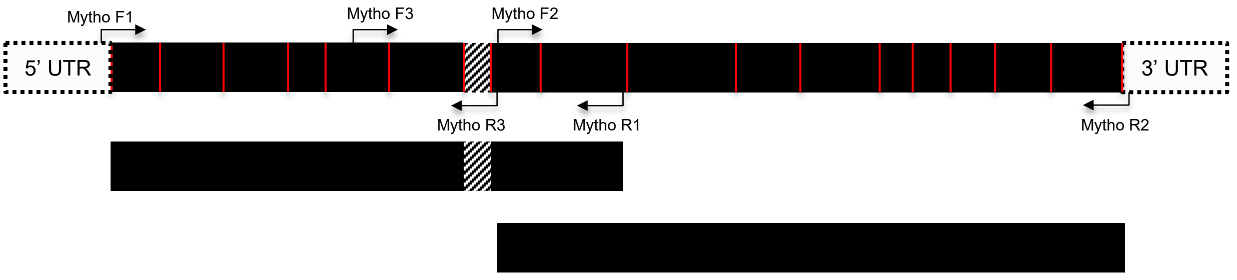

Figure S1: Schematic representation of the cloning strategy for *mytho* zebrafish cDNAs. Primers' position is indicated. *mytho* cDNA was amplified in two fragments overlapping in the central region. A diagonal hatching was used to shade the region present or absent in exon 7. White dotted rectangles correspond to the 5'- and 3'-untranslated regions.

```
            atctggcacagtcagagctcctcgttgatatccccatttttccccgcca
atgctggatctggaggtcgttcctgagcgatctttgggaaatgaacaatgggaattcgca
 M  L  D  L  E  V  V  P  E  R  S  L  G  N  E  Q  W  E  F  A
ttaggatgccattggcccaggccatttcaatattacagaaacactgccgaatcatcaaa
 L  G  M  P  L  A  Q  A  I  S  I  L  Q  K  H  C  R  I  I  K
aatgtccaggtgctctacagcgaacaaatgccactcagccacgacctcattctcaacttg
 N  V  Q  V  L  Y  S  E  Q  M  P  L  S  H  D  L  I  L  N  L
actcaggatggaattaaactgctgtttgatgcctgcaatcagagactgaaagtaattgaa
 T  Q  D  G  I  K  L  L  F  D  A  C  N  Q  R  L  K  V  I  E
gtgtacgatttgagcaaagtcaaactaaaatactgtggagtgcattttaactctcaggca
 V  Y  D  L  S  K  V  K  L  K  Y  C  G  V  H  F  N  S  Q  A
atagcacctactatcgagcagattgatcagtcttttggcgctactcatccaggagtctac
 I  A  P  T  I  E  Q  I  D  Q  S  F  G  A  T  H  P  G  V  Y
aatgctgccgagcagctcttccatctgaacttcagagggctttctttctccttccagctg
 N  A  A  E  Q  L  F  H  L  N  F  R  G  L  S  F  S  F  Q  L
gactcctggagtgaagccccctaaatatgagcctaactttgcccatggcttggcctccctg
 D  S  W  S  E  A  P  K  Y  E  P  N  F  A  H  G  L  A  S  L
cagaattcctcatggggccactgtcaaacggatgtacatctactccggaaacaaccttcaa
 Q  I  P  H  G  A  T  V  K  R  M  Y  I  Y  S  G  N  L  Q
gaaaccaaggctccagccatgcctctggcctgtttcttggtaatgtgtatgcagagtgt
 E  T  K  A  P  A  M  P  L  A  C  F  L  G  N  V  Y  A  E  C
gtggaggtgctgagagatggagcaggaccgctgggtctcaaacttcgtctcctcactgca
 V  E  V  L  R  D  G  A  G  P  L  G  L  K  L  R  L  L  T  A
ggatgcggcccaggagtgctggctgatactaaagtgcgagccgtagagagaagcatctac
 G  C  G  P  G  V  L  A  D  T  K  V  R  A  V  E  R  S  I  Y
tttggagattcctgtcaggatgttttgagtgctcttggctccccacataaggtcttctac
 F  G  D  S  C  Q  D  V  L  S  A  L  G  S  P  H  K  V  F  Y
aaatcagaggacaaaatgaagatccattcaccttcccctcacaagcaagtcccgtccaaa
 K  S  E  D  K  M  K  I  H  S  P  S  P  H  K  Q  V  P  S  K
tgtaacgactacttctttaactattatatactgggagtggatatactgtttgactcaaca
 C  N  D  Y  F  F  N  Y  Y  I  L  G  V  D  I  L  F  D  S  T
acccacctggtcaaaaaatttgtccttcacaccaacttccctggccattacaacttcaac
 T  H  L  V  K  K  F  V  L  H  T  N  F  P  G  H  Y  N  F  N
atatatcatcgctgtgatttcaagattcctctcatcattaaaaagatggagcggatgct
 I  Y  H  R  C  D  F  K  I  P  L  I  I  K  K  D  G  A  D  A
cacagtgaggactgcattttgaccacctacagcaaatgggatcagattcaggagctgctg
 H  S  E  D  C  I  L  T  T  Y  S  K  W  D  Q  I  Q  E  L  L
ggacaccctatggaaaaaccagttgtgctacacaggtcatcttctgcaaacaacgccaac
 G  H  P  M  E  K  P  V  V  L  H  R  S  S  S  A  N  N  A  N
ccttttggttccacgttttgttttggactgcagagaatgatatttgaggtcatgcagaac
 P  F  G  S  T  F  C  F  G  L  Q  R  M  I  F  E  V  M  Q  N
aaccacatagcttcagtgactctctacggagcaccacgtccaagtagtctggcccgcgtc
 N  H  I  A  S  V  T  L  Y  G  A  P  R  P  S  S  L  A  R  V
gagccgagtacccactgaggaagccgccatgcccacg
 E  P  S  T  H  -
```

Figure S2: *mytho* nucleotide coding and aminoacidic sequences. The underlined regions correspond to the primers that have been used to sequence the gene. In red are highlighted the starting and stop codons and the boundaries between different exons. In green are highlighted the differences in the nucleotide and aminoacidic sequences. The region highlighted in yellow corresponds to the thirty-three nucleotides that were missing from exon seven in our sequence, while the adenosine highlighted in violet represents the left boundary of the cryptic site for the alternative splicing.

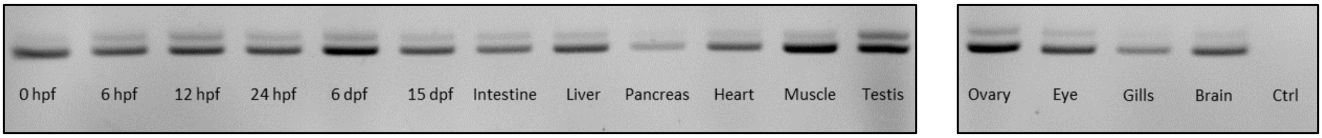

Figure S3: RT-PCR of *mytho* expression to highlight the presence of two isoforms of the transcript. Using primers that span the regions where we found the loss of thirty-three nucleotides, we observed that both transcript variants are present at different developmental stages and adult organs.

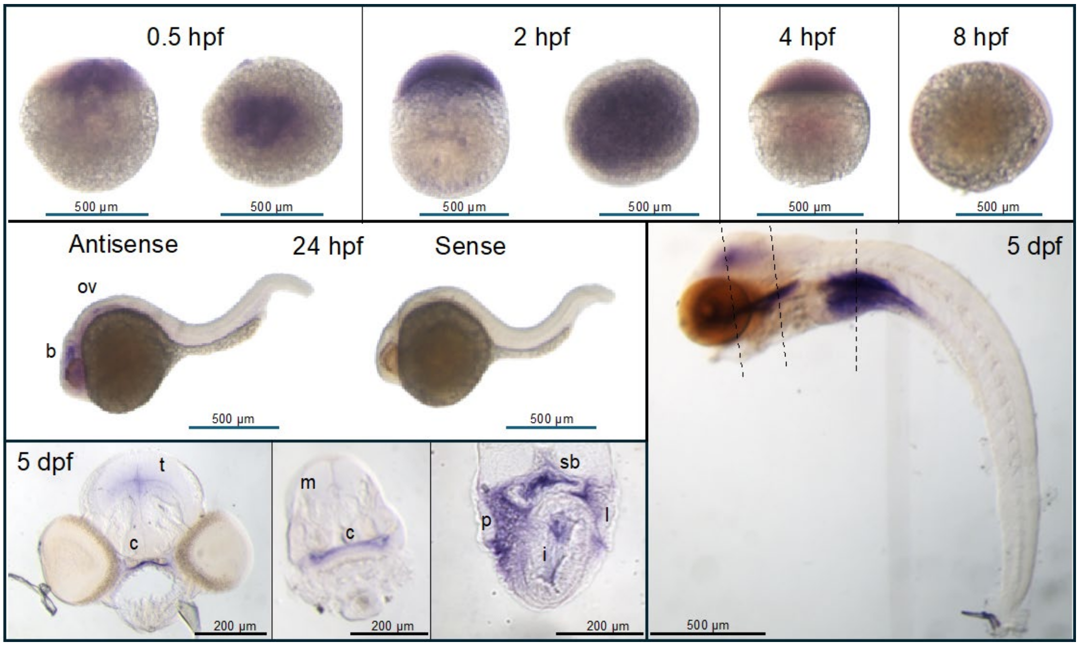

Figure S4. *mytho* whole mount *in situ* hybridization. A) Spatio-temporal expression of *mytho* mRNA at different development stages. Embryos at 0.5 and 2 hpf are lateral, with the animal pole up, and dorsal views; embryos from 8 hpf to 5 dpf larvae are lateral view and head up pointing to the left. B) Transverse vibratome sections of 5 dpf larvae. b = brain; c = chondrocranium; i = intestine; l = liver; m = mesencephalon; ov = optic vesicle; p = pancreas; sb = swim bladder; t = telencephalon.

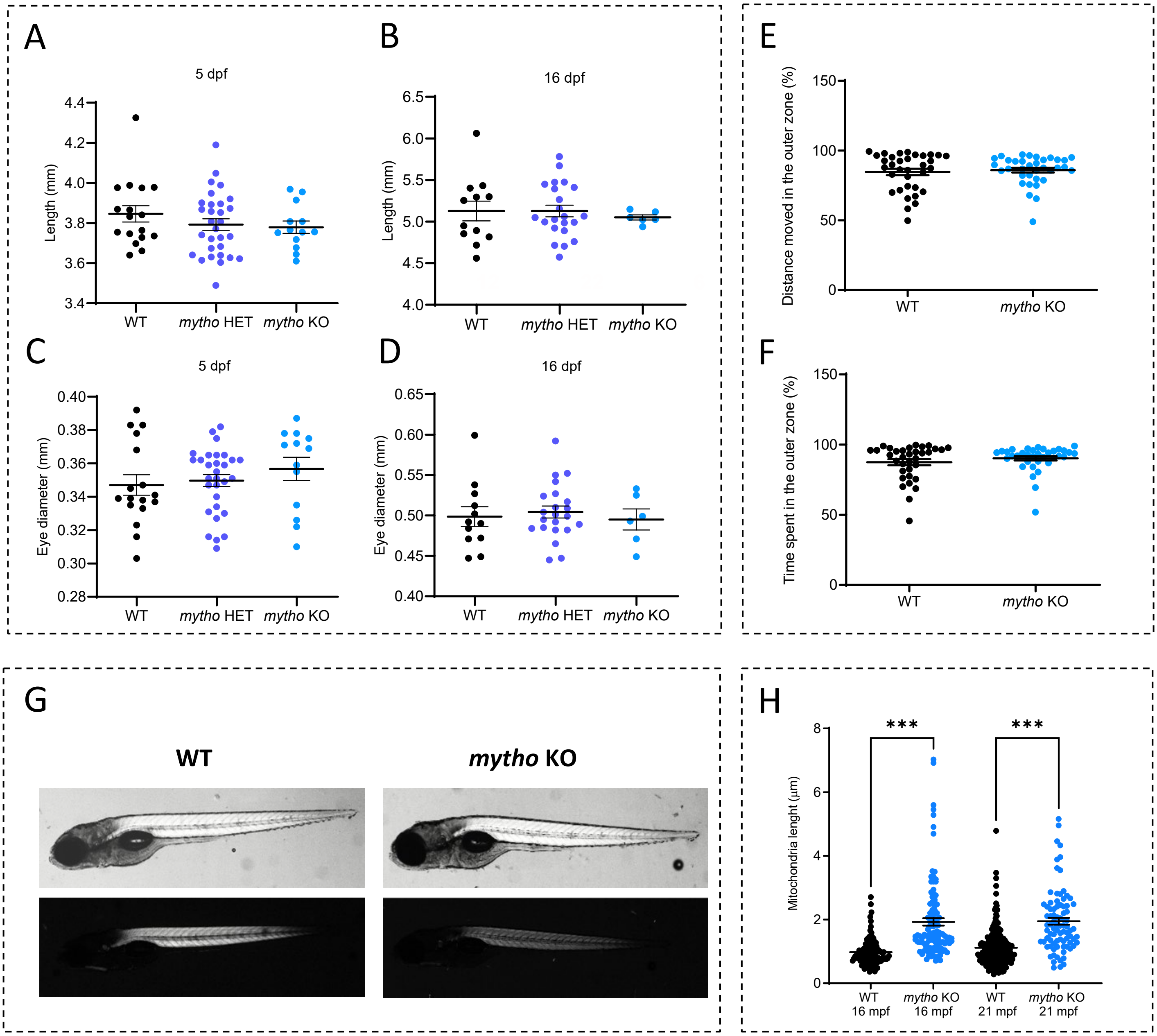

Figure S5. *mytho* KO larvae show no change in body feature sizes but present reduced locomotion and skeletal muscle defects.

A) length of 5 dpf larvae (in mm) of WT (n=17), heterozygous (n=30) and *mytho* KO (n=13). Statistical significance was tested with a general linear model ($F_{2,57}$=0.8881, p=0.417).

B) length of 16 dpf larvae (in mm) of WT (n=12), heterozygous (n=22) and *mytho* KO (n=6). Statistical significance was tested with a general linear model ($F_{2,37}$=0.1075, p=0.8984).

C) eye diameter of 5 dpf larvae (in mm) of WT (n=17), heterozygous (n=30) and *mytho* KO (n=13). Statistical significance was tested with a general linear model ($F_{2,57}$=0.7071, p=0.4973).

D) eye diameter of 16 dpf larvae (in mm) of WT (n=12), heterozygous (n=22) and *mytho* KO (n=6). Statistical significance was tested with a general linear model ($F_{2,37}$=0.1909, p=0.827).

E,F) Thigmotaxis test was performed in 5 dpf WT(n=34) and *mytho* KO (n=35) larvae. The percentage of distance moved (E) and time spent (F) in the outer zone of the plate which reflects the anxiety of the animals were calculated for WT and *mytho* KO. Statistical significance was tested with a general linear model ((E) $F_{1,67}$=0.0705, p=0.7915; (F) $F_{1,67}$=0.4527, p=0.5034).

G) Representative Images of skeletal muscle birefringence of 5 dpf larvae of WT and *mytho* KO zebrafish animals.

H) Analysis of mitochondrial length (in µm) using TEM images from adult (16 mpf) and old (21 mpf) WT and *mytho* KO animals. Number of mitochondria analyzed: WT (16 mpf) n=111; (21 mpf) n=184; *mytho* KO (16 mpf) n=110; (21 mpf) n=84. Statistical significance was tested with a linear model for the effect of genotype ($F_{1,485}$=173.1476, p<0.001) on mitochondrial length. P-values in post-hoc pairwise comparisons were adjusted using the Tukey method. ***p<0.001

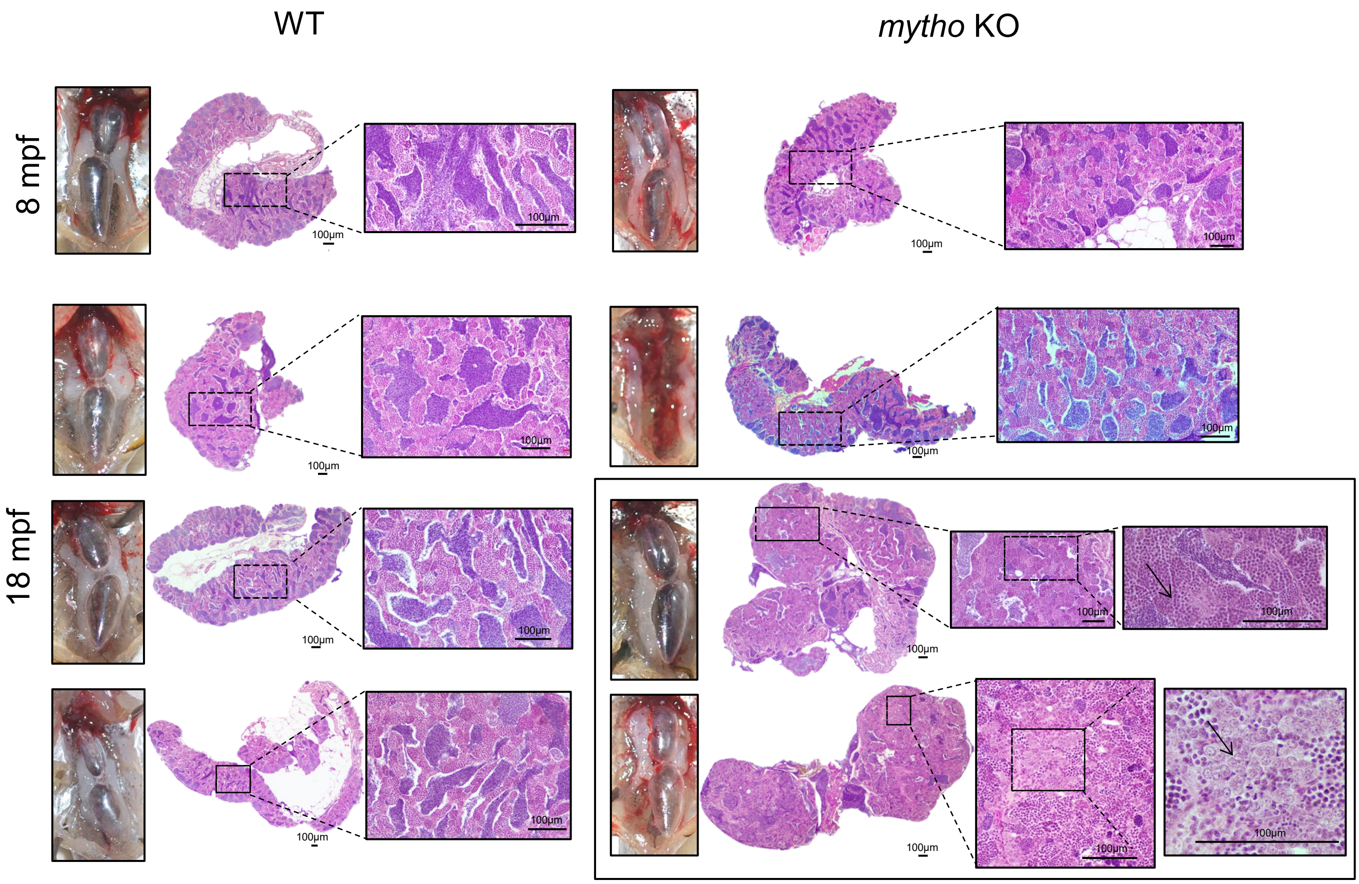

Figure S6. Histological sections of WT and *mytho* KO testicles at 8mpf and 18mpf with exacerbated alterations and the presence of seminomas in the aged *mytho* KO testis (lower right box).

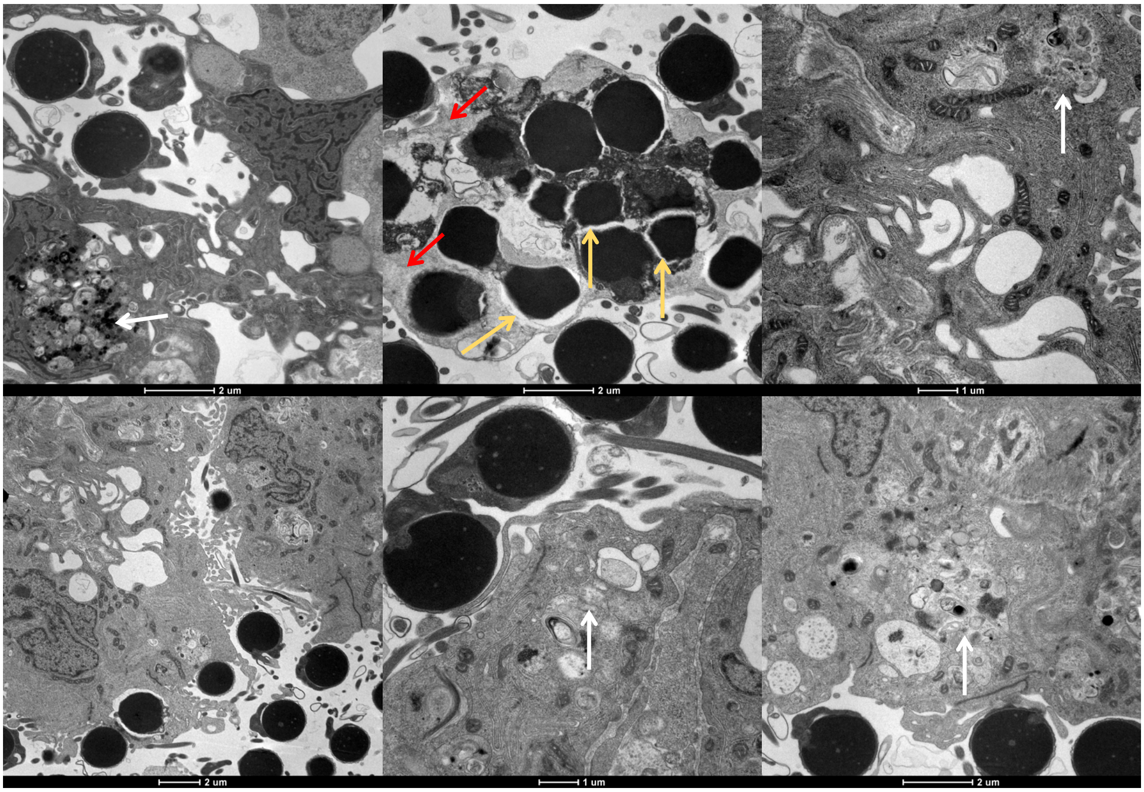

Figure S7. Example of a *mytho* KO testis with exacerbated alterations in testis morphology and spermatic cells**.** Perinuclear space (yellow arrows), residual cytoplasmic material (red arrows), and autophagic vesicles (white arrow) are found in this animal.

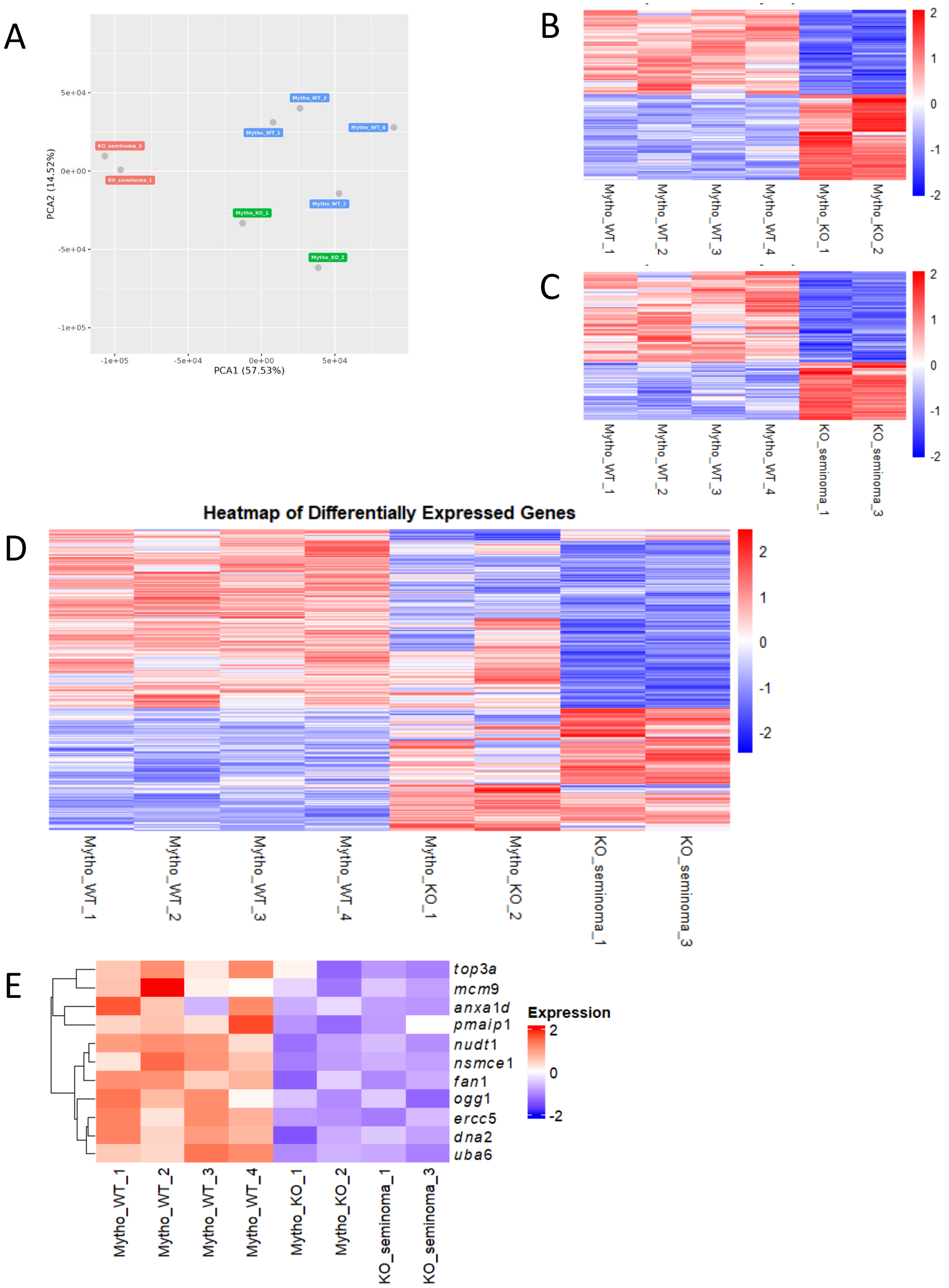

Figure S8-1. RNAseq analysis. A) Principal Component Analysis (PCA) conducted on the normalized gene expression values of the samples. X- and Y-axes show the PC1 and PC2, respectively, with the amount of variance explained by each component reported in parenthesis. Each point in the plot represents a sample, dots of the same colours are replicates of a same experimental group. **B,C,D)** Heat maps representing the expression patterns (Z-scaled raw counts) of Differentially Expressed Genes (DEG) in different comparisons. Columns correspond to samples and rows to DEGs. Changes in expression levels are displayed from blue (less expressed) to red (more expressed). The order of the genes was established after hierarchical clustering using Pearson correlation. E) Heatmap showing expression patterns (z-scaled raw counts) across the samples of DEGs related to genome stability maintenance downregulated in *mytho* KO testes. The genes were manually selected, only keeping the ones whose function has been verified experimentally or with a clear homology to other known genes. Columns correspond to samples and rows to DEGs. Changes in expression levels are displayed from blue (less expressed) to red (more expressed). Hierarchical clustering of the genes was established using Pearson correlation.

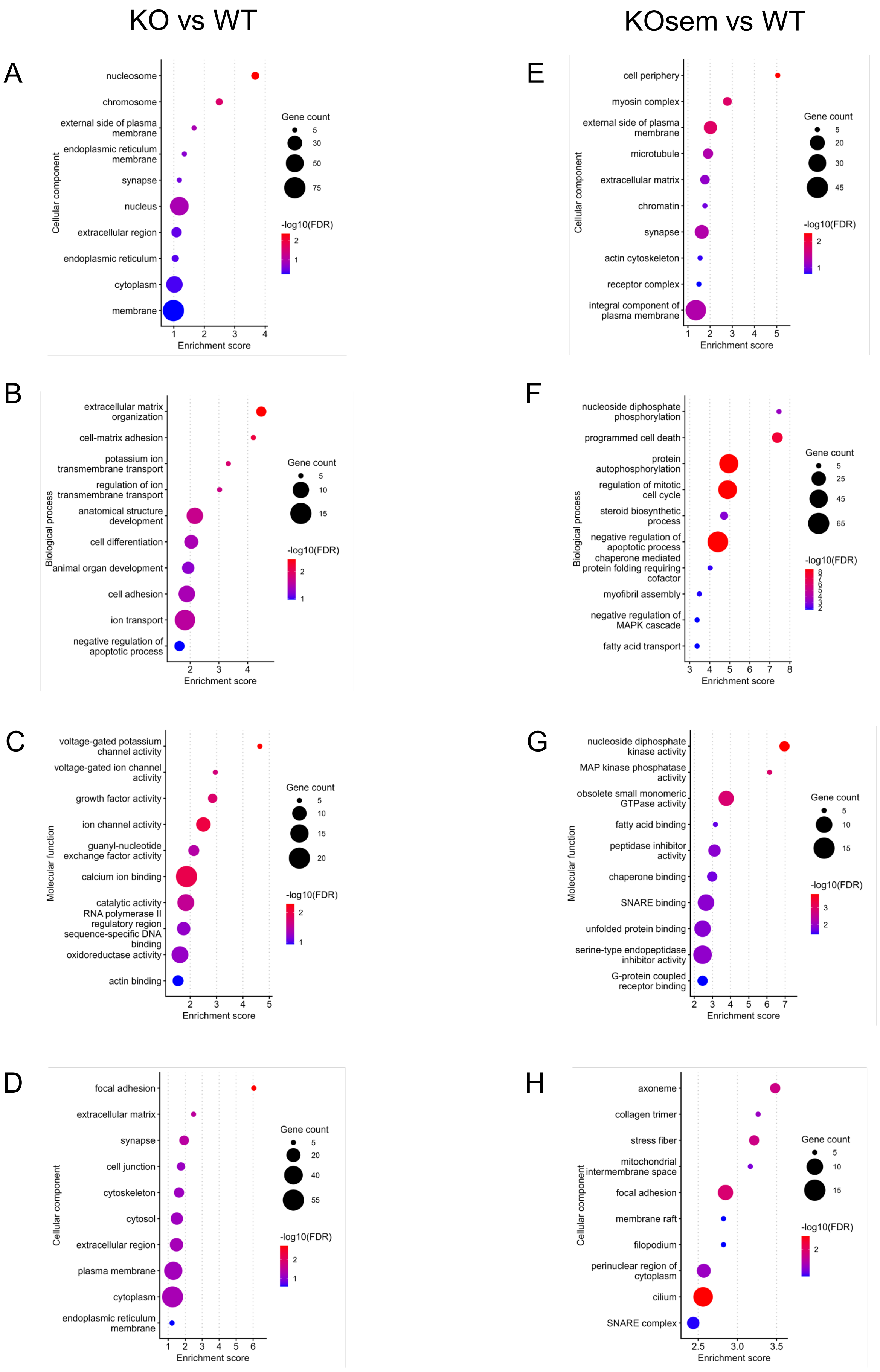

Figure S8-2. RNAseq analysis. Bubble plots representing gene ontology enrichment analyses (GOEAs) of the mytho KO vs WT (A-D) and seminoma-bearing mytho KO vs WT (E-H) comparisons. Biological Process, Molecular Function and Cellular component categories were considered. Only GOs represented by 5 or more genes were used for the analyses and the first ten GOs in term of Enrichment Score (reported in abscissa) are shown in the graph. Size and colour of the bubbles respectively represent the number of genes in each GO and the statistical significance of the enrichment

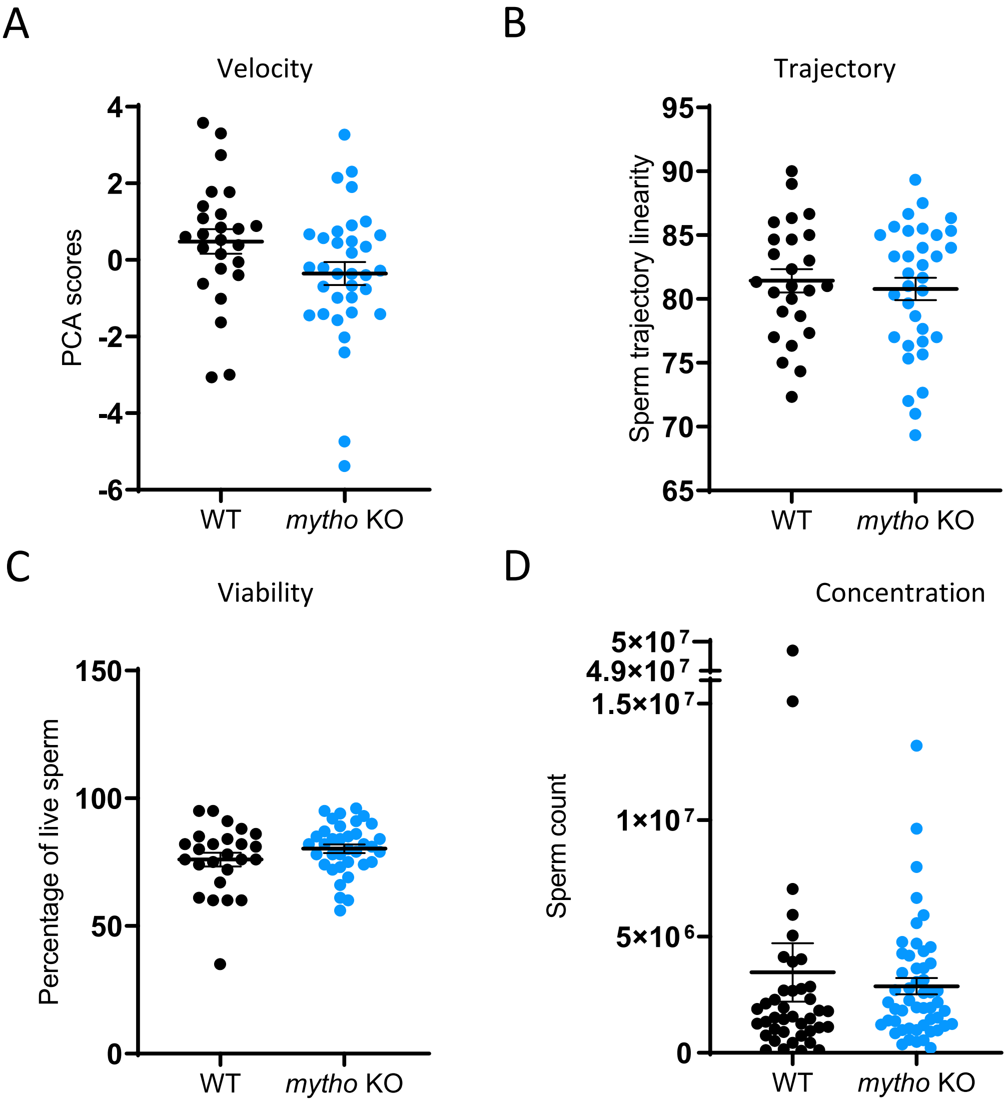

Figure S9. *mytho* KO sperms have unaltered velocity, trajectory, viability and concentration compared with WT.

A) Sperm velocity traits (based on 1000 parametric bootstraps). Since sperm velocity parameters (VAP, VSL, VCL) were all highly correlated (all Pearson r > 0.96) a combined index of sperm velocity was generated from the 3 velocity variables using a principal component analysis (PCA). The scores of the first component, accounting for 98.54% of variance and with all sperm traits showing similar positive loadings, were used for the analyses on sperm velocity (N: Mytho KO=18, WT=17). Statistical significance was tested with a linear model ($F_{1,33}$=1.118, p=0.298).

B) Linearity of sperm trajectory (LIN) based on 1000 parametric bootstraps (N: Mytho KO=18, WT=17). Statistical significance was tested with a linear model ($F_{1,33}$=0.465, p=0.5).

C) Sperm viability was compared between WT and MYTHO KO (N: Mytho KO=20, WT=17). Statistical significance was tested with a beta regression model (χ 2=1.1645, df=1, p=0.2805).

D) Number of sperm was counted between WT and *mytho* KO animals (N: Mytho KO=20, WT=17). Statistical significance was tested with a linear model ($F_{1,35}$=0.408, p=0.527).

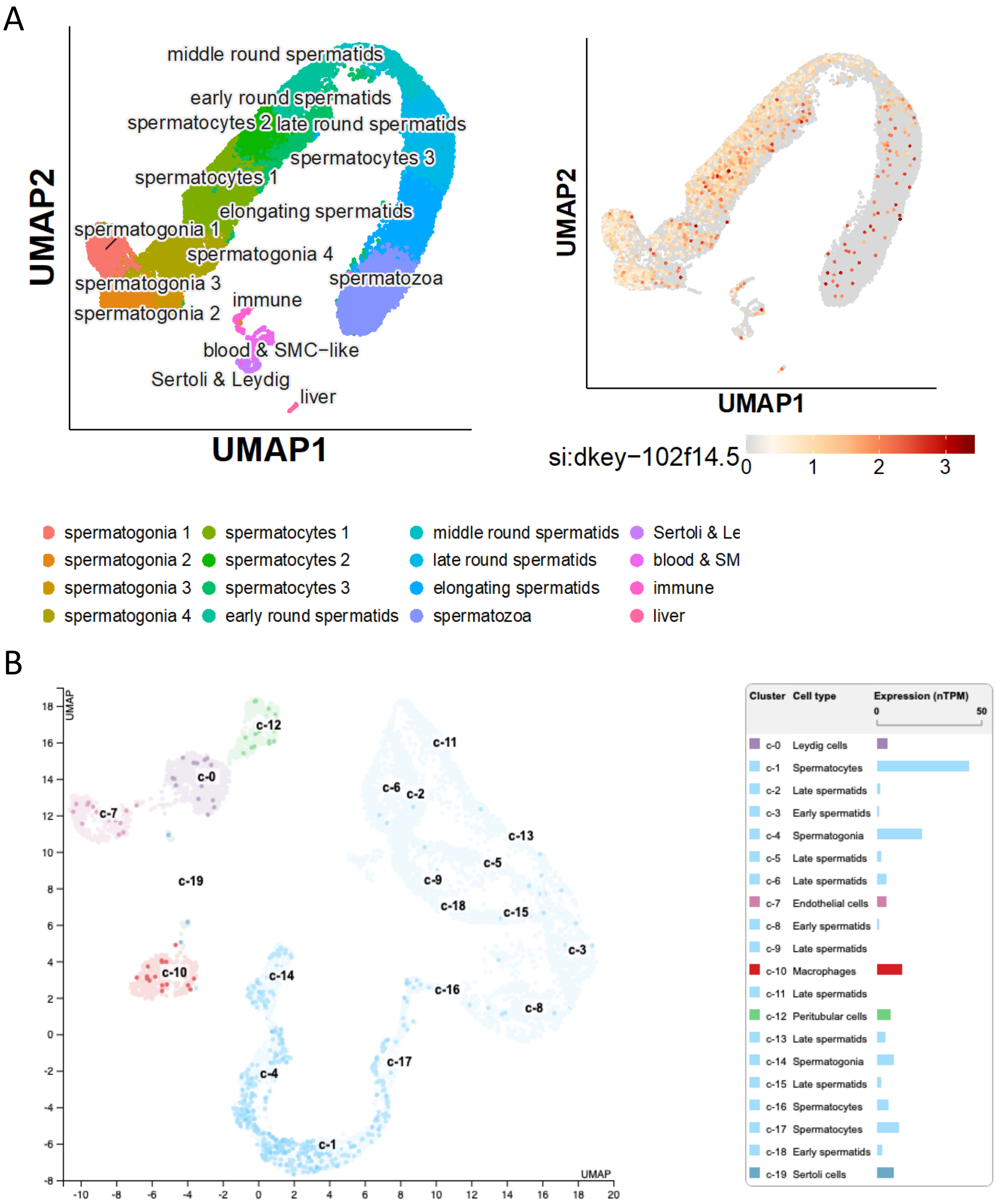

Figure S10. *mytho* is more expressed in zebrafish and human spermatogonia and spermatocytes while its expression is lower in early and late spermatids. A) Image of the si:dkey-102f14.5 (*mytho*) expression in zebrafish testis cell types showing the higher expression of this gene in spermatogonia and spermatocytes. The analysis was performed with a web-based application (https://github.com/asposato/zebrafish_testis_fertility) that allows to explore single-cell datasets of zebrafish testis. B) Image from proteinatlas.org showing higher expression in c-14, c-4, c-1, c-17 (spermatogonia and spermatocytes) than in c-8, c-3, c-15, c-9, c-18, c-5, c-13, c-2, c-6, c-11 (early and late spermatids).

Supplemental Tables

Table S1. Summary tables of histological alterations of male and females gonads, retina, pancreas and skeletal muscles from WT and *mytho* KO animals of different ages. The table A provides an overview of the type and frequency of alterations observed in *mytho* KO animals. The Table B provides a detailed breakdown of these observations by age and sex. Number of animals analyzed are specified in the table. NAD: non-alteration-detected.

A

	Gonads	Pancreas	Retina	Muscle
Male WT	Testicular hyperplasia **2/11**	NAD	Retina degeneration (ganglion cell layer) **1/11**	NAD
Male KO	Seminoma **5/9**	Pancreatic degeneration **1/12**	Retina degeneration (ganglion cell layer) **6/12**	Degeneration and necrosis **2/12**
	Tubular ectasia **1/9**			
	Testicular degeneration **1/9**			
Female WT	Focal ovarian degeneration **3/6**	NAD	NAD	NAD
	Multifocal ovarian degeneration **2/6**			
Female KO	Focal ovarian degeneration **5/9**	Pancreatic degeneration **2/9**	Retina degeneration (ganglion cell layer) **4/9**	Degeneration and necrosis **4/9**
	Ovarian retention **2/9**			
	Immature ovary **2/9**	Pancreatic carcinoma **1/9**		
	Granulomatous oophoritis **6/9**			

B

MALES	Number of tested subjects	Gonads	Pancreas	Retina	Skeletal muscle
mytho WT 11 mpf	5	NAD	NAD	NAD	NAD
mytho WT 15 mpf	2	NAD	NAD	1/2 degeneration of retina (ganglion cell layer)	NAD
mytho WT 17 mpf	4	2/4 testicular hyperplasia	NAD	NAD	NAD
mytho KO 6 mpf	1	NAD	NAD	degeneration of retina (ganglion layer)	NAD
mytho KO 11 mpf	2	1/2 tubular ectasia/testicular degeneration	NAD	1/2 degeneration of retina (ganglion layer)	NAD
mytho KO 13-15 mpf	3	1/3 seminoma	NAD	NAD	NAD
mytho KO 16-17 mpf	3	2/3 seminoma	NAD	2/3 degeneration of retina (ganglion layer)	1/3 degeneration and necrosis
mytho KO 20-22 mpf	3	2/3 seminoma	1/3 pancreatic degeneration	2/3 degeneration of retina (ganglion cell layer)	1/3 degeneration and necrosis

FEMALES	Number of tested subjects	Gonads	Pancreas	Retina	Skeletal muscle
mytho WT 4 mpf	1	NAD	NAD	NAD	NAD
mytho WT 12 mpf	3	2/3 focal ovarian degeneration<br>1/3 multifocal ovarian degeneration	NAD	NAD	NAD
mytho WT 20 mpf	2	1/2 focal ovarian degeneration<br>1/2 multifocal ovarian degeneration	NAD	NAD	NAD
mytho KO 5 mpf	1	Ovarian retention, and degeneration	Pancreatic degeneration	degeneration of retina (ganglion cell layer)	degeneration and necrosis
mytho KO 12- 17 mpf	4	4/4 focal ovarian degeneration and granulomatous oophoritis<br>1/4 ovarian retention	1/4 pancreatic carcinoma	1/4 degeneration of retina (ganglion cell layer)	1/3 degeneration and necrosis
Mytho KO 19-23 mpf	4	1/4 ovarian degeneration<br>2/4 immature ovary<br>2/4 granulomatous oophoritis	1/4 pancreatic degeneration	2/4 degeneration of retina (ganglion cell layer)	2/4 degeneration and necrosis

Table S2. Principal component analyses on sperm velocity parameters.

	**Principal component loadings**		
	PC1	PC2	PC3
**1 VAP**	0.581	0.120	0.805
**2 VSL**	0.576	0.637	-0.511
**3 VCL**	0.574	-0.761	-0.301
**Eigenvalues**	2.956	0.042	0.001
**Total variance (%)**	98.54	1.42	0.05
**Cumulative variance (%)**	98.54	99.95	100

Table S3. Within sample repeatability for sperm velocity (VAP, VSL, VCL), linearity of sperm trajectory (LIN), sperm mobility and sperm viability. Reported are the point estimate R, standard error SE, confidence interval CI and P values.

	**R**	**SE**	**CI**	**P (LRT)**
**VAP**	0.721	0.088	0.497 - 0.847	4.32e-07
**VSL**	0.719	0.089	0.493 - 0.844	4.81e-07
**VCL**	0.661	0.103	0.416 - 0.812	6.93e-06
**LIN**	0.324	0.145	0.008 - 0.608	0.0314
**motility**	0.745	0.085	0.529 - 0.868	1.13e-07
**viability**	0.266	0.139	0 - 0.517	0.0618

Table S4. CRISPR target site oligonucleotides (37).

Oligo name	Sequence
**Constant oligo**	AAAGCACCGACTCGGTGCCACTTTTTCAAGTTGATAACGGACTAGCCTTATTTTAACTTGCTATTTCTAGCTCTAAAAC
**mytho specific oligo**	ATTTAGGTGACACTATA**TGATCAATCTGCTCGATAGT**GTTTTAGAGCTAGAAATAGCAAG

* ***PCR*** *Bold and italic sequence correspond to gRNA*

Table S5. Primers/oligos used in this article to amplify *mytho* for different purposes.

Technique	primer name	Sequence
Sequencing	*mytho* F1	atctggcacagtcagagctc
	mytho R1	tgaggagacgaagtttgagac
	mytho F2	cactgtcaaacggatgtacatc
	mytho R2	cgtgggcatggcggcttc
In situ probe	*mytho* F2	cactgtcaaacggatgtacatc
	mytho R2	cgtgggcatggcggcttc
q-PCR	*mytho* F2	cactgtcaaacggatgtacatc
	mytho R1	tgaggagacgaagtttgagac
	ube2a F1	catcatggtctggaacgctg
	ube2a R1	gaggaaacgtcatatgttggac
Alternative splicing RT-PCR	*mytho* F3	ctactatcgagcagattgatcag
	mytho R3	gatgtacatccgtttgacagtg
Genotyping	*mytho* F4	ctattgctctgttcattctagtg
	mytho R4	caactgaccctagcaatgaatc
