# Supplementary material for: Mytho/Phaf1 is required to prevent DNA damage and tissue degeneration in Danio rerio
Source: Cell Death Discov. 2026 Apr 17;12:252. doi: 10.1038/s41420-026-03106-x (PMC13212566; doi:10.1038/s41420-026-03106-x)

Original membrane of fig. 1E blot – Mytho expression

Markers

Mytho

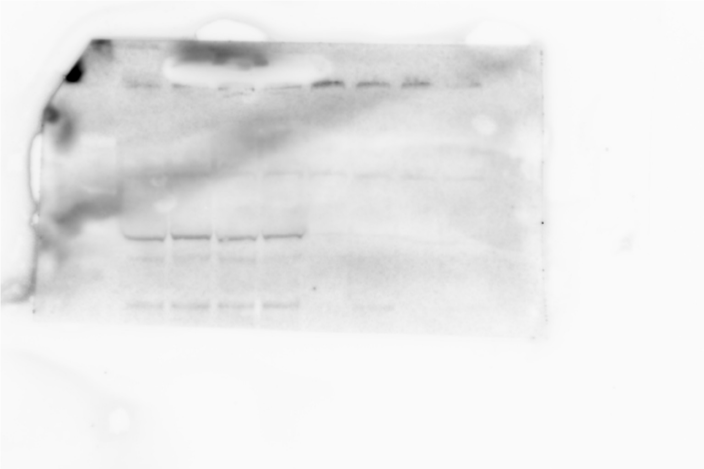

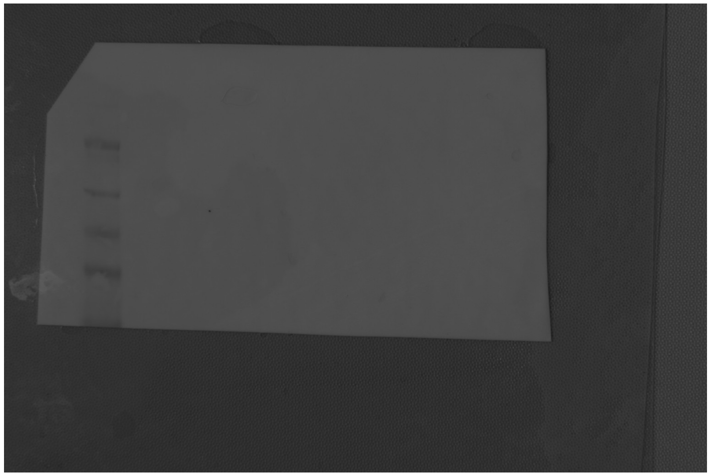

β - Actin

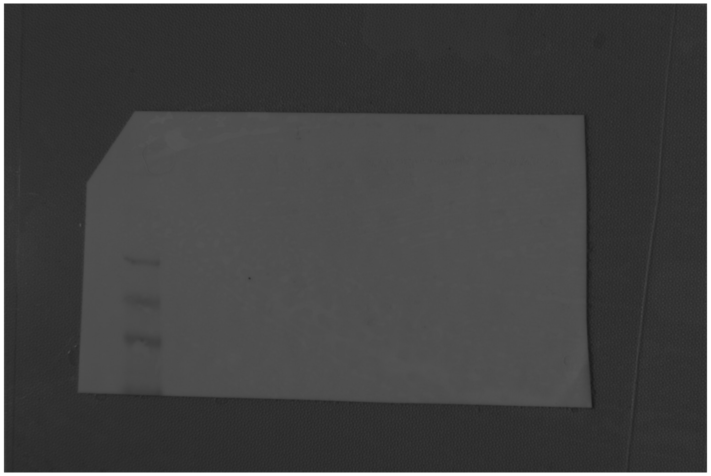

Original membrane of fig. 3B blot – LC3 expression

This membrane was used to represent the blot but does not show all the samples

Markers

LC3II

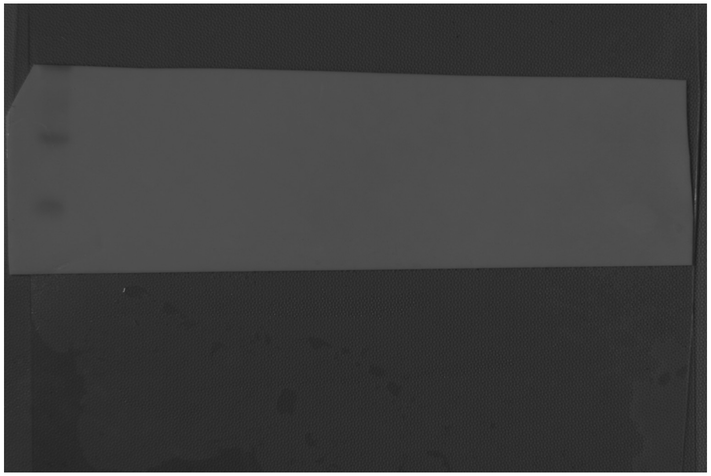

β - Actin

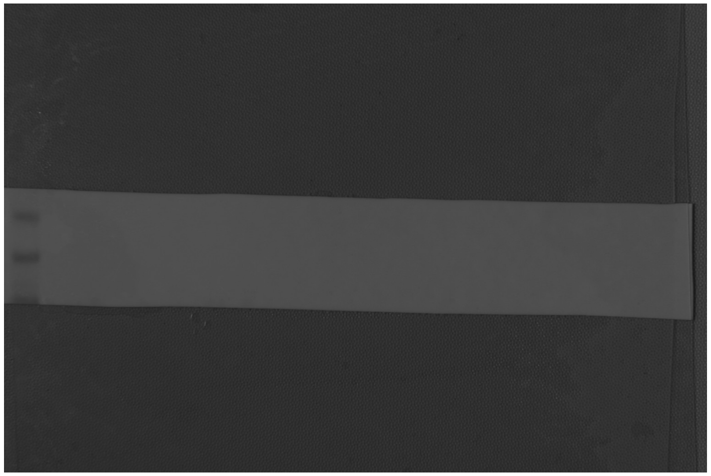

Original membranes of fig. 3B blot – LC3 expression

Here are shown both membranes used for the quantification. The lighter one is the same of the previous slide

Markers

LC3II

β - Actin

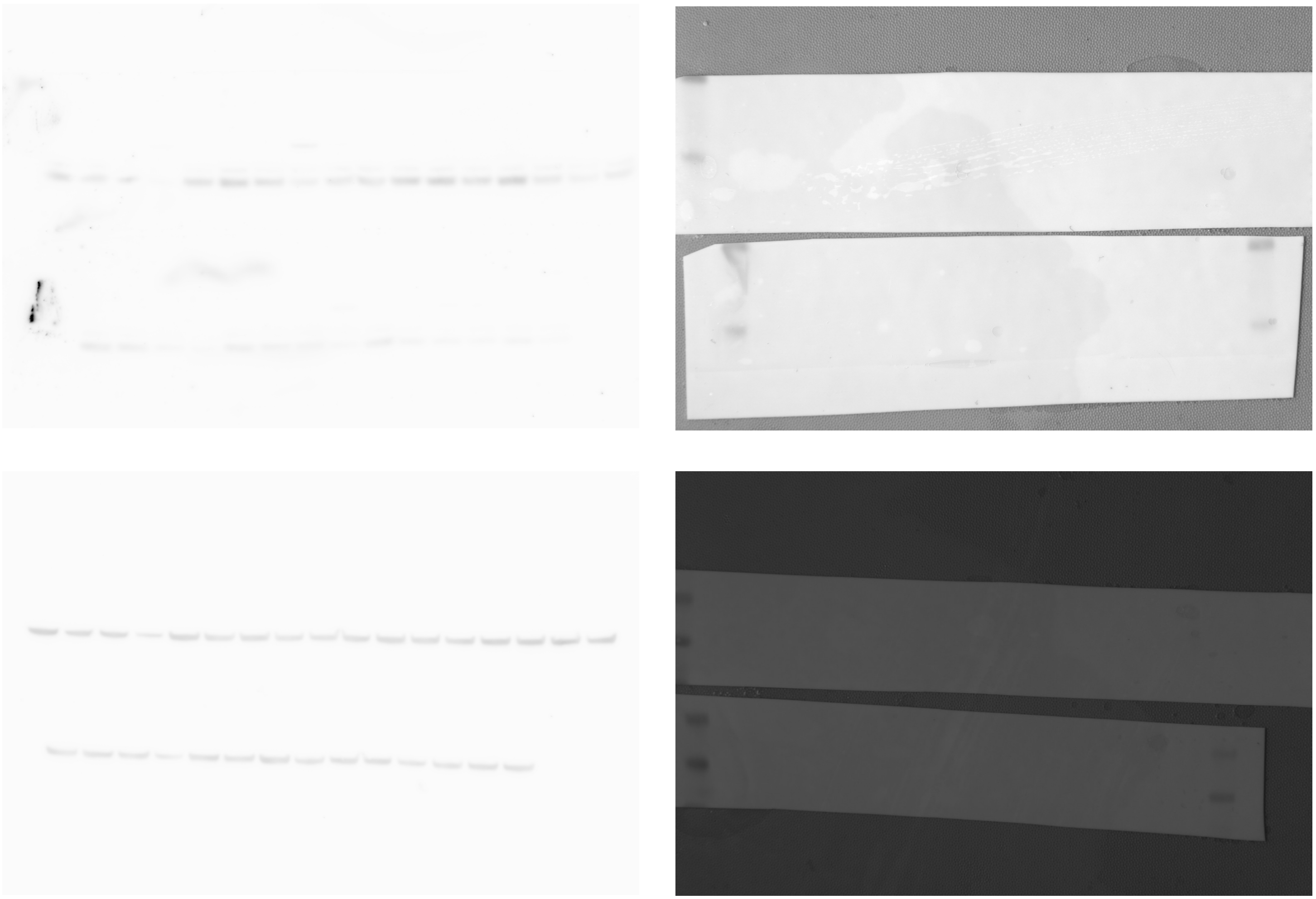

Supplement: Supplementary file 6 — Uncropped blots [file 41420_2026_3106_MOESM6_ESM.pdf]
